# Ethnopharmacology of Medicinal Plants of the Pantanal Region (Mato Grosso, Brazil)

**DOI:** 10.1155/2012/272749

**Published:** 2012-02-26

**Authors:** Isanete Geraldini Costa Bieski, Fabrício Rios Santos, Rafael Melo de Oliveira, Mariano Martinez Espinosa, Miramy Macedo, Ulysses Paulino Albuquerque, Domingos Tabajara de Oliveira Martins

**Affiliations:** ^1^Department of Basic Sciences in Health, Faculty of Medicine, Federal University of Mato Grosso, Cuiabá, Avenida Fernando Correa da Costa, No. 2367, University Campus, 78060-900 Cuiabá, MT, Brazil; ^2^Department of Statistics, Institute of Exact and Earth Sciences, Federal University of Mato Grosso, 78060-900 Cuiabá, MT, Brazil; ^3^Faculty of Biological Sciences, University of Cuiabá, Mato Grosso, Avenida Beira Rio, No. 3100, University Campus, 78020-590 Cuiabá, MT, Brazil; ^4^Department of Biology, Botany Area, Laboratory of Applied Ethnobotany, Federal University of Rural de Pernambuco, 52171-900 Recife, PE, Brazil

## Abstract

Traditional knowledge is an important source of obtaining new phytotherapeutic agents. Ethnobotanical survey of medicinal plants was conducted in Nossa Senhora Aparecida do Chumbo District (NSACD), located in Poconé, Mato Grosso, Brazil using semi-structured questionnaires and interviews. 376 species of medicinal plants belonging to 285 genera and 102 families were cited. Fabaceae (10.2%), Asteraceae (7.82%) and Lamaceae (4.89%) families are of greater importance. Species with the greater relative importance were *Himatanthus obovatus* (1.87), *Hibiscus sabdariffa* (1.87), *Solidago microglossa* (1.80), *Strychnos pseudoquina* (1.73) and *Dorstenia brasiliensis*, *Scoparia dulcis* L., and *Luehea divaricata* (1.50). The informant consensus factor (ICF) ranged from 0.13 to 0.78 encompassing 18 disease categories,of which 15 had ICF greater than 0.50, with a predominance of disease categories related to injuries, poisoning and certain other consequences of external causes (ICF  =  0.78) having 65 species cited while 20 species were cited for mental and behavioral disorders (ICF  =  0.77). The results show that knowledge about medicinal plants is evenly distributed among the population of NSACD. This population possesses medicinal plants for most disease categories, with the highest concordance for prenatal, mental/behavioral and respiratory problems.

## 1. Introduction

Despite the fact that modern medicine, on the basis of the complex pharmaceutical industry, is well developed in most part of the world, the World Health Organization (WHO) through it Traditional Medicine Program recommends its Member States to formulate and develop policies for the use of complementary and alternative medicine (CAM) in their national health care programmes [1]. Among the components of CAM, phytotherapy practiced by the greater percentage of the world population through the use of plants or their derivatives, occupies a significant and unique position [2].

In this sense, documentation of the indigenous knowledge through ethnobotanical studies is important in the conservation and utilization of biological resources [3].

Brazil is a country with floral megadiversity, possessing six ecological domains, namely, Amazonian forest, Caatinga, Pampas, Cerrado, Atlantic Forest, and the Pantanal [4]. Mato Grosso region is noteworthy in this regard, as it occupies a prominent position both in the national and international settings, for it presents three major Brazilian ecosystems (the Pantanal, Cerrado, and Amazonian rainforest). Besides this, it also hosts diverse traditional communities in its territories, namely, the Indians descents (Amerindians), African descents, and the white Europeans. However, due to the mass migration from the rural areas and technological development, coupled with globalization of knowledge by the dominant nations, cultural tradition concerning the use of medicinal plants is in the major phase of declining [5].

The Pantanal is distinguishably the largest wetland ecosystem of the world, according to the classification by UNESCO World Heritage Center (Biosphere Reserve) [4]. The Pantanal vegetation is a mosaic consisting of species of the Amazonian rainforest, Cerrado, Atlantic forest, and Bolivian Chaco, adapted to special conditions, where there is alternations of both high humidity and pronounced dryness during the time of the year [4]. The presence in the Pantanal of the traditional populations that use medicinal plants for basic health care makes this region an important field for the ethnobotanical and ethnopharmacological studies [6, 7].

Because of the fact that the Pantanal communities are relatively isolated, they have developed private lives that involved much reliance on profound knowledge of the biological cycles, utilization of natural resources, and traditional technology heritage [8].

As a result of the aforementioned, this study aimed to systematically and quantitatively evaluate the information gathered from these Pantanal communities, highlight the relevance of the ethnobotanical findings, and cite and discuss relevant literatures related to medicinal plants with greater relative importance (RI) and high informant consensus factor (ICF) values obtained in the study.

## 2. Materials and Methods

### 2.1. Study Area

**Figure 1:**
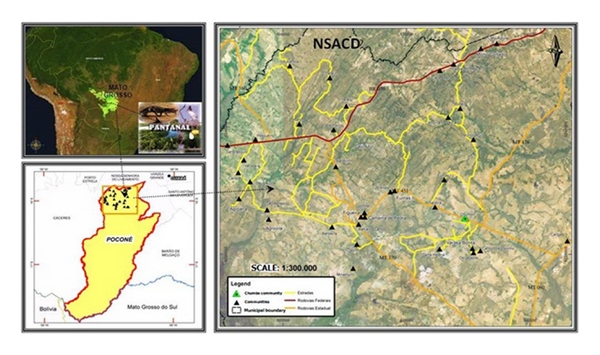
Location of the study area. Poconé, Mato Grosso, in Midwest of Brazil.

For the choice of study area, literature search was conducted to identify the Pantanal region in Mato Grosso, consisting of traditional communities where such studies have not yet been conducted and/or there were no ethnobotanical survey publications. The study design was cross-sectional and was conducted between the period of November, 2009 and February, 2010. The study setting chosen was NSACD located in the Poconé municipality, Mato Grosso State, Central West of Brazil (Figure 1) with coordinates of 16° 02′ 90′′ S and 056° 43′ 49′′ W. Poconé is located within the region of Cuiabá River valley, with an altitude of 142 m, occupies a territorial area of 17,260.86 km^2^, and of tropical climate. The mean annual temperature is 24°C (4–42°C) and the mean annual rainfall is 1,500 mm with rainy season occurring between December and February. The municipality is composed of 2 Districts (NSAC and Cangas), 5 villages, 11 settlements, 14 streets, and 72 communities (countryside) [9]. The population of NSACD is estimated to be 3,652 inhabitants, representing 11.5% of Poconé municipality [10]. The principal economic activities are mainly livestock farming, mining, and agriculture with great tourism potentials, because Poconé municipality is the gateway to the Pantanal region [9].

### 2.2. Consent and Ethical Approval

Authorization and ethical clearance were sought from the relevant government (Health authority of Poconé and the National Council of Genetic Heritage of the Ministry of Environment (CGEN/MMA), Resolution 247 published in the Federal Official Gazette, in October, 2009, on access to the traditional knowledge for scientific research and Federal University of Mato Grosso and Júlio Muller Hospital Research Ethical Committees, Protocol 561/CEP-HUJM/08 authorities. Previsits were made to each community of NSACD to present the research project as well as to seek the consent of each potential informant.

### 2.3. Data Collection and Analysis

In this present study, sampling was done using probabilistic simple randomization and stratified sampling techniques [10, 11].

The population studied consists of inhabitants of 13 communities of NSACD, Mato Grosso State, considering an informant per family. The criteria for each informant chosen were age of 40 and above, residing in NSACD for more than 5 years (because there is large migration into the area because of the presence of ethanol producing factory).

These criteria are in line with the study objective coupled with the information gathered from the local authority [12].

In order to determine the estimated sample size (*n*), in this case, the number of families to be sampled per communities being considered, the following formula was utilized [11, 13]:

(1) $$n=\frac{Np\left(1-p\right)}{\left(N-1\right){\left(d/z_{\alpha /2}\;\right)}^{2}+p\left(1-p\right)}.$$

This study considered the population size of 1,179 families (*N* = 1, 179), confidence coefficient of 95% (*z*/2 = 1.96), sampling error of 0.05 (*d* = 0.05), a proportion of 0.5 (*P* = 0.5). It should be noted that the *P* = 0.5 was assigned due to nonexistence of previous information about this value as is usual in practice, to obtain conservative sample size which is representative at the same time.

In determining the sample size for the microarea, 5% error and 10% loss in sample were considered. To determine the sample size in each microarea, the sample size (290) was multiplied by the sampling fraction of each microarea and dividing the total number of families of the same microarea with the total number of families of all the microareas (1,179), thereby arriving at the sample sizes for each area as shown in Table 1.

The interviews were conducted with the help of 12 trained applicators, under the supervision of the respective investigator. Data collected included sociodemographic details, vernacular names of the plant species with their medicinal uses, methods of drug preparation, and other relevant information. The ethnobotanical data were organized using the Microsoft Office Access 2003 program and statistically analyzed using SPSS, version 15 for Windows (SPSS Inc., Chicago, Illinois, USA).

### 2.4. Plant Collection, Identification, and Herborization

The collection of plant materials were done in collaboration with the local specialists, soon after the interviews. Both indigenous and scientific plant names were compiled. The plant materials collected during the study period were herborized, mounted as herbarium voucher specimens, and deposited for taxonomic identification and inclusion in the collection of Federal University of Mato Grosso and CGMS Herbarium of Federal University of Mato Grosso do Sul, Brazil.

Plant species were identified according to standard taxonomic methods, based on floral morphological characters, analytical keys, and using, where possible, samples for comparison, as well as consultations with experts and literature [6, 7, 14–19]. The plant species obtained were grouped into families according to the classification system of Cronquist [20], with the exception of the Pteridophyta and Gymnospermae. For corrections of scientific names and families, the official website of the Missouri Botanical Garden was consulted [21].

### 2.5. Quantitative Ethnobotany

The relative importance (RI) of each plant species cited by the informants was calculated according to a previously proposed method [22]. In order to calculate RI, the maximum obtainable by a species is two was calculated using (2) according to Oliveira et al. [23]

(2) RI = NCS + NP,

where RI: relative importance; NCS: number of body systems. It is given by the number of body systems, treated by a species (NSCS) over the total number of body system treated by the most versatile species (NSCSV): NCS = NSCS/NSCSV; NP: number of properties attributed to a specific species (NPS) over the total number of properties attributed to the most versatile species (NPSV): NP = NPS/NPSV.

We sought to identify the therapeutic indications which were more important in the interviews to determine the informant consensus factor (ICF), which indicates the homogenity of the information [23].

The ICF will be low (close to 0), if the plants are chosen randomly, or if the informants do not exchange information about their uses. The value will be high (close to 1), if there is a well defined criterion of selection in the community and/or if the information is exchanged among the informants [23].

ICF was calculated using the number of use citations in each category of plant disease (*n*_ur_), minus the number of species used (*n*_*t*_) divided by the number of use citations in each category minus one on the basis of (3):

(3) $$\text{ICF}=\frac{n_{\text{ur}}-n_{t}}{n_{\text{ur}}-1}.$$

The citations for therapeutic purposes were classified using the 20 categories of the International Classification of Diseases and Related Health Problems, 10th edition-CID [24]: injuries, certain infectious, and parasitic diseases (I); neoplasms-tumors (II), diseases of blood and blood-forming organs and certain disorders involving the immune mechanism (III), endocrine, nutritional and metabolic diseases (IV) mental and behavioral disorders (V), nervous system (VI), diseases of eye and adnexa (VII), diseases of the ear and mastoid process (VIII), diseases of the circulatory system (IX), respiratory diseases (X), digestive diseases (XI), diseases of the skin and subcutaneous tissue (XII), diseases of the musculoskeletal system and connective tissue (XIII), genitourinary diseases (XIV), pregnancy, childbirth and (XV), certain conditions originating during the perinatal period (XVI), symptoms, signs and abnormal clinical and laboratory findings, not elsewhere classified (XVIII) and injury, poisoning and certain other consequences of external causes (XIX).

We selected for further discussion species that presented RI ≥ 1.5, and are in a category with high ICF. We conducted literature review using among others, the databases of Web of Science, MEDLINE, SciELO and including nonindexed works. We also searched national data bases for dissertations and theses.

## 3. Results

A total of 262 informants were interviewed, representing 7.17% of the population of NSACD, 22.22% of the population aged ≥40 years and residing in the District for over five years. Of the respondents, 69% were female and 31% male, aged 40–94 years (median 55). 68% were born in the city of Poconé, and 62% have been residents for over 20 years in the District (Table 2).

Of the 262 respondents, 259 (99.0%) reported the use of medicinal plants in self health care, with a minimum of 1 plant and a maximum of 250 plants among the female respondents and a minimum of 2 plants and a maximum of 54 among the male respondents. A total of 3,289 citations were recorded corresponding to 376 different plant species which belong to 285 genera and 102 families. Fabaceae (10.2%), Asteraceae (7.82%), and Lamaceae (4.89%) families were the most representative in this study (Table 3).

### 3.1. Relative Importance (RI)

The RI of the species cited by 262 respondents from NSACD ranged from 0.17 to 1.87. A total of 261 species had RI ≤ 0.5; 80 species, RI from 0.51 to 1.0; 30 species, RI from 1.1 to 1.5, and 4 species with RI from 1.51 to 2.0, among the latter, three species were native to Brazil. The species with RI ≥ 1.5, were *Himatanthus obovatus *(Müll. Arg.) Woodson (1.87),* Hibiscus sabdariffa* L. (1.87),* Solidago microglossa *DC. (1.80),* Strychnos pseudoquina *A. St.-Hil. (1.73),* Dorstenia brasiliensis *Lam.,* Scoparia dulcis *L., and *Luehea divaricata* Mart. (1.50 each), as shown in Table 4.

### 3.2. Informant Consensus Factor (ICF)

In the disease categories according to CID, 10th ed., we observed that ICF values ranged from 0.43 to 0.77, with the exception of disease category included in CID VI (diseases of the nervous system), which was 0.13. The ICF for CID VI ranged between 0.13 and 0.78 (mean = 0.62, SD = 0.16, 95% CI: 0.53–0.70). The highest consensus value obtained was for the category related to injuries, poisoning, and some other consequences of external causes (ICF = 0.78), with 65 species and 286 citations. Three species were more common, namely, *S. dulcis* and *S. microglossa* (“Brazilian arnica”), with 49 citations each and *L. pacari* (manga-brava) with 42 citations. The main ailments addressed in this category were inflammation, pain, and gastric disorders.

Out of 20 disease categories, there were citations for 18 therapeutic indications, as shown in Table 5.

## 4. Discussion

In the present study, almost all the respondents (99%) claimed to know and use medicinal plants. Surveys conducted in other countries had reported values ranging from 42% to 98% depending on the region and country of the study [25–27]. Due to the low level of knowledge of traditional medicine in national capitals, ethnobotanical surveys in many developing countries including Brazil, primarily prefer to evaluate small communities or rural hometowns, whose population having knowledge and practical experience with traditional medicine are proportionately higher (between 80 and 100%) [28–30].

The high percentage of folk knowledge of medicinal plants identified in Brazil may be due to factors such as lower influence of the contemporary urban lifestyle and the strength of cultural traditions in the rural communities [31]. In fact, with the process of industrialization and migration to the cities, a significant part of traditional culture is maintained more in the communities farther from the metropolis via oral transmission of the knowledge of CAM and family traditions. Transmission and conservation of CAM knowledge is more pronounced in Brazil due to high degree of biodiversity.

One of the most important aspects of this research is the documentation of high number of taxa (285 genera and 102 families) and species (376) mentioned by the informants as medicinal. These findings confirmed the existence of the great diversity of plants used for therapeutic purpose and preserved traditional culture, as stated by Simbo [32]. It is worth mentioning here the presence of 8 (eight) local medicinal plant expert informants/healers among the 262 respondents in this study. These local expert informants/healers account for a significant number of citations (43 to 250) in this study. In Brazil, as in other countries, rural communities have developed knowledge about the medicinal and therapeutic properties of natural resources and have contributed to the maintenance and transmission of the ethnopharmacological knowledge within the communities.

The most representative plant families are Fabaceae (10.2%), Asteraceae (7.82%), and Lamiaceae (4.89%). These results are in accordance with other ethnobotanical surveys conducted in the tropical regions [33, 34] including Brazil [7, 35]. Furthermore, the results from our study are also in conformity with the findings of the most comprehensive ethnobotanical survey conducted by V. J. Pott and A. Pott in the Brazilian Pantanal region [19].

Featuring greater potential for bioprospecting are 231 (61.6%) species indicated for the treatment of at least two diseases, and RI between 0.17 and 1.87 (mean = 0.46, SD = 0.357, 95% CI: 0.4250–0.4973). The seven species with the highest RI were *H. obovatus *(Müll. Arg.) Woodson (13 therapeutic indications and RI = 1.87,* H. sabdariffa *L. (12 therapeutic indications and RI = 1.87); *S. microglossa* DC. (9 therapeutic indications and RI = 1.80) *S. pseudoquina* A. St. - Hil. (14 therapeutic indications and RI = 1.73) and *D. brasiliensis* Lam., *S. dulcis *L., and *L. divaricata* Mart. (12, 10, and 12 therapeutic indications respectively with RI = 1.50) (Table 4). For the sake of brevity, we will focus most of our discussion on these seven most cited medicinal plants highlighting the most important available literature on them and including *L. pacari*. It should be noted that although 146 (39%) species presented RI below 0.17, with just a single indication, they cannot be considered as of lower pharmacological potential or importance, because as Albuquerque et al. [36] have noted elsewhere, these may be species of recent introduction in the culture of the community under study but might have been validated by the customary use in other social groups.

A total of 105 different folkways, including 18 disease categories, according to Brasil [24], were codified as shown in Table 5. The highest frequencies in decreasing magnitude were indications for the treatment of pain and inflammation (10.8%), kidney disease (7.6%), and wound healing (6.8%). In part, these data can be explained by the characteristics of the informants (elderly, rural activity, low level of education, and poor sanitation at home) with higher frequency of chronic, inflammatory, and infectious diseases. In addition, the search for natural treatments for infected wounds is very common in populations of agrarian labor or menial worker as stated by Akerreta et al. [37]. As ICF values were generally close to 1.0, it may be presumed that there is certain homogeneity in knowledge of medicinal plants among the population of NSACD.

### 4.1. Literature Survey and Discussions on the Selected Species with Higher Relative Importance

*Himatanthus obovatus*, var.* obovatus *had the highest relative importance, being cited for 13 different ailments that fall into 11 categories of CID, 10th ed. with a total of 29 citations. The most commonly mentioned of these indications for this plant were its traditional use as a blood cleansing, wound healing, and other conditions associated with infections, which seems to point to its possible antibiotic activity. Indeed, some studies have demonstrated the *in vitro* activity of its different extracts against promastigotes of *Leishmania donovani *[38]. A few others also showed experimentally its antiviral, antitumor activities, cellular proliferation activities, and inflammatory and immune response [39, 40]. On the basis of these aforementioned, it is possible that its use in the folk medicine may be related to its ability to modulate the immune system, which may enhance physiological mechanisms involved in resolving inflammation, pain, and wound healing. We did not encounter any literature pertaining to its use in anemia, nosebleeding, muscle relaxant, deworming, or vitiligo treatment. Its indications as a blood cleansing and as antihypercholesterolemic are important targets for future biomedical research.

*Hibiscus sabdariffa *calyces are used in many parts of the world to make cold and hot drinks as well as in folk medicine [41]. Due to its many health-enhancing benefits, extensive works have been carried to validate its traditional therapeutic claims. In fact, its medicinal importance is widely acknowledged in many traditional herbal systems [42].

The benefits associated with the use of *H. sabdariffa* may in part be due to its high content of beneficial phytochemical constituents. These include alkaloids, L-ascorbic acid, anisaldehyde, anthocyanin, *β*-carotene, *β*-sitosterol, citric acid, cyanidin-3-rutinoside, delphinidin, galactose, gossypetin, hibiscetin, mucopolysaccharide, pectin, protocatechuic acid, polysaccharide, quercetin, stearic acid, and flavonoids [42, 43]. Studies have highlighted the role of polyphenol acids, flavonoids, and anthocyanins that may act as antioxidants or through other mechanisms that may contribute to its cardioprotective activity [44, 45].

In additions to folkloric use of *H. sabdariffa* noted in this study, other previous reports have indicated its use in the treatment of liver disease, hypocholesterolemic, antispasmodic, intestinal antiseptic, sedative, and as mild laxative [42, 46]. The most extensively studied is its antihypertensive activity. This effect was confirmed in several *in vitro *and animal studies [47–49]. The hypotensive effect of* H. sabdariffa *and its constituents may be mediated, at least partially, by a cholinergic and/or histaminergic mechanism and it has been confirmed to act via inhibitiory action on angiotensin I converting enzyme, vasorelaxation [50], and diuretic action [51]. For detailed review on this aspect, see [41]. In addition to literature reports on the medicinal uses of this plant, we also report here its indications in the treatment of anxiety and labyrinthitis and as anti-snake venom. To the best of our knowledge, these indications remained to be proven experimentally.

In concordance with the traditional use of *H. sabdariffa* in the treatment of uterine inflammation and pain, its aqueous ethanol extract was shown experimentally to presents anti-inflammatory, uterine antispasmodic activities, and attenuation of intestinal spasm [52–54]. In addition to its confirmed pharmacological activities, its antiobese/weight-reducing [50, 55], hepatoprotective [56–58], anticancer [46, 59, 60], free-radical scavenging [61], antioxidant [42], immunomodulatory [62], lipid-lowering [43, 63] effects and attenuation of oxidants-mediated complications in diabetes [64] have been well documented. Besides, the plant extract is characterized by a very low degree of toxicity [41]. Moreover, apart from its medicinal uses, the plant seed oil was also shown to be a good source of lipidsoluble antioxidants, particularly **γ**-tocopherol, thus it could have important industrial applications [65].

*Solidago microglossa *is popularly known in Brazil as “arnica,” “arnica-do-mato,” “arnica-silvestre,” “erva-federal,” “arnica-vulgar,” “erva-lanceta,” and “rabo-de-rojão” [66]. It is usually confused with *Arnica montana* L., a native of the mountainous regions of Europe, due to the similarity in their medicinal flowers and having the same color (yellow), *S. microglossa *is not cultivated in Brazil due to it low adaptation to the tropical conditions [66]. In our study, *S. microglossa* was indicated for treatment of 15 different diseases corresponding to 8 classes of CID, 10th ed. and had a total of 49 citations. The key citations for this plant were its use in wound healing and blood cleansing. Other popular indications found in this study were similar to those previously reported, especially its use in the treatment of wounds, acne, bruises, and stomach-related ailments [67].

Several classes of compounds and metabolites have been isolated from *S. microglossa*, especially phenols, acetophenones, carotenoids, lactones (helenalin and dihydro-helenalin) [68, 69], flavonoids [70, 71] saponins [72], and polyacetylenes [70]. The cicatrizant activity of the plant's extract has been confirmed experimentally [73]. Although not mentioned directly by respondents in this study, some lines of evidence suggest important antibiotic activity with the use of *S. microglossa*, which can justify its indication for uterine inflammation. Morel et al. [74] showed that the essential oil of *S. microglossa* and three of its components (quercetrin, *α*-espinasterol, and solidagenone) are capable of significantly inhibiting the growth of *Staphylococcus aureus*,* Staphylococcus epidermidis*,* Klebsiella pneumoniae*,* Escherichia coli*,* Salmonella setubal*,* Bacillus subtilis*,* Pseudomonas aeruginosa*,* Saccharomyces cerevisiae, *and* Candida albicans *[74]. In addition, cicatrizant activity was observed with the administration of the plant's extract [73]. Its use in ameliorating renal ailments, blood cleansing, and hypotensive and antiparasitic activities may be associated with the presence in high concentrations of tannins [75, 76] and flavonoids in this species [76–79]. Its indication for muscle relaxation may also derive from its antispasmodic effect [80]. Further studies are warranted in these regards.

Other pharmacological properties not mentioned here, but have been established in preclinical studies, include hypoglycemic effect [81] and antitumor activity. In fact, the latter effect has attracted intense interest in the discovery of new chemotherapeutic agents. The extract of *S. microglossa* demonstrated antiproliferative effect (but not mutagenic) against young shoot cells of onion (*Allium cepa*) strain [82]. Some of these activities may be related to the presence of secondary metabolites such as helenalin [83].

Although *Strychnos pseudoquina* is referred to locally as “quinas”, similar to the local name used for species such as *Cinchona* sp. (source of quinine), it has been shown to be inactive against *Plasmodium berghei* [84] contrary to its popular use in folk medicine elsewhere [84]. Theoretically, some of the indications may result from the classification bias in the community due to an erroneous popular cultural belief that plants referred to as “quinas” are useful for “anemic” patients infected with malaria parasite. This perhaps helps to explain why the highest indication for this plant in our study was to treat anemia.

Among the components isolated from *S. pseudoquina* metabolites are isoramnetin, strychnobiflavone, and 11-diaboline metoxidiaboline [85]. Silva et al. [86] demonstrated the gastroprotective effect of *S. pseudoquina* in models of gastric lesions induced by nonsteroidal anti-inflammatory agents and some necrotizing agents, thus confirming its indication for gastric ulcer and stomach disorders as noted in this present study. On the other hand, its indication in wound healing has not been experimentally confirmed at least in the diabetic wound model in rats [87] or in local hemorrhage induced by *Bothrops jararaca* venom [88]. Other medicinal uses indicated like “blood depurative” and analgesic effect may be subject of future investigation as a potential agent with antinociceptive and metabolic disorders ameliorating effects. Regarding its toxicity, Santos et al. [81] showed that only the methanol extract (but not dichloromethane) from the leaves of *S. pseudoquina* have mutagenic effect in *Salmonella* strains TA98 (−S9) and TA100 (+ S9, −S9) and that it induces formation of micronuclei after acute treatment [81].

*Dorstenia brasiliensis*, known as “Carapiá” is a perennial herb of the early geological point of view, typical of the fields in southern Brazil, Paraguay, Uruguay, and Argentina [89, 90]. Phytochemical analysis of roots of *D. brasiliensis* indicated the presence of dorstenic acid A and B (triterpenoids), isopimarane-type diterpenoid, and six different types of coumarins. The two triterpenoids showed moderate cytotoxicity against leukemia cells (L-1210 and HL-60) [91]. Furthermore, some authors have suggested that its use in cutaneow disease (such as psoriases and vitiligo) may be associated with the presence of furanocoumarins in the species of *Dorstenia* [92]. Bartericin A and B, stigmasterol, isobavachalcone, 4-hydroxylonchocarpin, dorsmanin F, 6,8-diprenyleridictyol, quercetin, quercitrin, amentoflavone [93], psoralen, bergapten (from rhizome), and umbelliferone [94] are some of the compounds isolated this medicinal plant.

Some few pharmacological studies have demonstrated analgesic and anti-inflammatory activities of *D. brasiliensis* in animal models [95]. These data corroborated the popular use of *D. brasiliensis* as an analgesic. There is dearth of information confirming its use in the popular medicine use as an anti-inflammatory agent. Moreover, *D. brasiliensis* may possesses some biologically active compounds similar to other *Dorstenia* species from the same genus and may thus share similar pharmacological profile. The following compounds and pharmacological activities have been reported in other *Dorstenia* species: chalcones (*D. prorepens* and *D. zenkeri*) [96], furocoumarins (*D. bahiensis* and *D. bryoniifolia*), triterpenes (*D. bahiensis, D. bryoniifolia*,* D. carauntae, D. cayapiaa, *and* D. heringerii*) [97]. This is a point to be noted for future research. Some authors have investigated its potential use as antivenom, antiinfective, anti-rheumatic [96, 97] while others established its antitrichomonal [93], antitussive [98], antioxidant [93, 99] and antileishmanial [100] activities.

*Scoparia dulcis*, popularly known as “vassourinha”, grows wild in backyards, gardens, and fields in Brazil. Phytochemical studies have identified the presence of more than 12 interesting pharmacologically active compounds in this species, namely, scoparic acid A [101], iso-dulcinol, 4-epi-scopadulcic acid B, dulcidiol, scopanolal, dulcinol/scopadulciol, scopadiol [102], scoparinol [103], scopadulcic acid B [104–106], glutinol [107] and scopadulin [105]. Scopadulcic acid B inhibited the effects of tumor promoter 12-O-tetradecanoylphorbol-13-acetate (TPA) *in vitro *and* in vivo*, and also suppressed the promoting effect of TPA on skin tumor formation, demonstrating stronger effect than antitumor-promoting terpenoids, such as glycyrrhetinic acid [104]. In fact, its cytotoxicity has been investigated against antitumor activity [102] and nerve growth factor-mediated neurite outgrowth and neurodegenerative disorders [103, 108].

The analgesic and anti-inflammatory activities of ethanol extracts of *S. dulcis* and glutinol have been demonstrated in writhing induced by acetic acid and carrageenan-induced paw edema, respectively [107]. However, *S. dulcis* extracts were ineffective in the central pain models (tail flick) and paw edema induced by dextran. Another secondary metabolite, scoparinol, also showed significant analgesic and anti-inflammatory activity [109]. In regard to its toxicological effects, it is worthwhile to mention that glutinol and scoparinol markedly potentiated pentobarbital-induced sedation and duration of sleeping time in these two studies mentioned above.

In contrast to its toxicity, *S. dulcis* seems to possess potential hepatoprotective activity in different models, which have been attributed to its free-radical scavenging potential activities [110–113]. Corroborating with antibiotic use for some infections (like gonorrhea), some authors have investigated inhibition of multidrug resistance (MDR) bacteria, fungi [114, 115], leishmanial parasite [116], and herpes simplex virus type 1 growths [96].

Paradoxically, despite the low citation in gastric ulcer and diabetes treatments in this study, the antiulcer and antihyperglycemic activities of this species are well documented. Inhibitory activities of *S. dulcis* extracts was demonstrated in pylorus ligature model, histamine- or bethanechol-stimulated gastric secretion, and acute gastric lesions induced by indomethacin [117, 118]. *S. dulcis* was also demonstrated to inhibit both proton pump (H^+^, K^+^-ATPase) and proton transport into gastric vesicles [105]. In regard to its antihyperglycemic effect, experimental evidences demonstrated that *S. dulcis *extracts reduced blood glucose, glycosylated haemoglobin, prevented decrease in the body weight, and improved glucose tolerance similarly with glibenclamide [119]. Even in the insulin resistance stage, *S. dulcis*-treated L6 myotubes were found to be more capable of stimulating glucose transport than insulin treatment [120]. In addition, scoparic acid D was able to stimulate insulin secretion and receptor binding in streptozotoci- (STZ-) induced diabetic rats [121].

*Luehea divaricata *is a native tree of the Brazilian Cerrado popularly known as “açoita-cavalo”. Just as popularly indicated, some studies have reported the following pharmacological activities of *L. divaricata*: the leaves as used as diuretic, the stems as anti-inflammatory, the bark and aerial parts are used for healing skin wounds, pimples, and for vaginal washes [122, 123].

Phytochemical screening of *L. divaricata* reported the presence of flavonoids, tannins and saponins and afforded the presence of 3b-*p*-hydroxybenzoyl-tormentic acid [124], maslinic acid [122], vitexin and glucopyranosylsitosterol, and (−)-epicatechin [123].

The presence of flavonoids and metabolites such as the vitexin [125, 126] and maslinic acid [127, 128] may be associated with the popular indication of its anti-inflammatory properties formation of urate (18) and antitumor (4). Extracts of *L. divaricata *has been shown to have antioxidant activity and analgesic property [129], lack toxicity *in vivo* [130], or mutagenicity [131]. Its extract also showed cytotoxicity against tumor cell lines [123]. Due to the high level of citation for the treatment of urate aleviation (18), we believe that its antigout or uricosuric activity may be an important target of pharmacological interest. Another indication prominently cited by the respondents is the use of *L. divaricata* in the treatment of lung diseases and upper airway. However, there is no scientific evidence on its regulatory activity on cough, while its antibiotic properties also vary. Some authors have demonstrated its inhibitory effect on the growth of dermatophytes [132] but not in other fungi species [123, 129]. In addition, the extract of *L. divaricata* was shown to strongly inhibit the growth of *S. aureus, S. epidermitis, K. pneumonia, *and* E. coli *in a study [129] but showed only moderately in another study elsewhere [123].

It is worth mentioning that although *Lafoensia pacari* A.St.-Hil. had low relative importance value, all the same, it is among the three plants with the highest informant consesus factor in addition to being a native plant in the region. The other two (*S. dulcis* and *S. microglossa*) have been discussed previously.

*L. pacari *popularly called “mangava-brava”, belongs to the family Lythraceae, is a tree native to the Brazilian Cerrado [133]. It is commonly used for gastrointestinal disorders, wound healing, diarrhea, and kidney problems. In our study, it was referenced for the treatment of seven disorders distributed into five classes of CID, 10th ed. Preliminary phytochemical studies of methanol extract of the stem bark of *L. pacari *revealed the presence of free steroids, saponins, tannins catechins, pyrogalic tannins (in particular, ellagic acid), triterpenoids, simple phenols, strong and weak fixed acids, alkali, and quaternary amino acids [134–136]. Acute toxicity studies or subchronic oral administration of extracts of *L. pacari* did not indicate any harmful effects [137]. However, it is also indicated for its adverse reactions and used as an abortifacient, diarrheic, weight loss, and tachycardia. Among the 42 citations for *L. pacari*, 29 were for the treatment of ulcer, and four and two for gastritis and stomach, respectively. These indications have been confirmed with the use of methanol crude extract of *L. pacari* and its major active components, ellagic acid, in different experimental ulcer models [138–143]. In addition, the antiulcer activity of the methanol extract (capsules) of *L. pacari* was confirmed in the clinical trial with 55 patients with dyspepsia [144].

We did not encounter any studies concerning its activities in wound healing, antidiarrheal or alleviation of kidney disorders. This phenomenon of plant selection by local people for certain indications may be, for instance, to consolidate best practice of the medicinal properties of the plants at the expense of using other plants substitute for these indications. In fact, the broad community access to Amazon or Pantanal biome, and the close relationship with the indigenous native populations, promotes a variety of possibilities of ethnobotanical indications. Examples of other popular uses of *L. pacari* that have been experimentally confirmed includes weight loss [145], anorectic effect [142], antipyretic activity [146], anti-inflammatory [147], antiallergic [148], and analgesic property [149].

It is also worth mentioning other studies focused on the medicinal uses of *L. pacari*, including its potent antifungal activity [150], have demonstrated that the main compound responsible is found in the methanol extract of this plant. A patent application of lotion with the infusion prepared from the leaves of *L. pacari*, as a component of the formulation was also solicited [151]. To the best of our knowledge, there is currently no available literature concerning its claims as wound healing, antidiarrheal, or in kidney disorders.

## 5. Conclusions

The present study identified the several plant species and their medicinal uses in NSACD highlighting significant cultural diversity in the Pantanal region. In fact, one of the important components of this community is the contribution of Amerindian culture, which highlights its importance in the identification of indigenous popular knowledge relevance in the identification of native popular knowledge.

Analytically, the data were categorized according to the highest values of relative importance and consensus among informants, ensuring the best evidence for ethnobotanical bioprospecting of medicinal plants. Thus, we identified seven native species with the highest relative importance, which are *H. obovatus*,* H. sabdariffa*, *S. microglossa*, *S. pseudoquina* and *D.brasiliensis*, *S. dulcis, *and* L. divaricata *including* L. pacari. *The three plants with the highest value of consensus among informants were *S. dulcis*, *S. microglossa,* and *L. pacari.*

The preservation of local culture, the practice of traditional medicinal plant species themselves represent important strategies for sustenance of popular knowledge of CAM in the local systems of health care and environmental education. Moreover, ethnobotanical and pharmacological studies provide information essential for guidance in bioprospecting for new drugs of plant origin in the consolidation of therapeutic practices of the community.

## Figures and Tables

**Table 1:** Distribution of the 13 communities of Nossa Senhora Aparecida do Chumbo District.

ID	COMMUNITY	Total number of individuals	Total number of families	Sample fraction	Sample size
1	Chumbo	946	216	0.1832	52
2	Canto do Agostinho, Santa Helena, Os Cagados, Várzea bonita	179	52	0.0441	15
3	Furnas II, Salobra, Zé Alves	165	59	0.0500	15
4	Campina II, Furnas I, Mundo Novo, Rodeio	279	81	0.0687	20
5	Campina de Pedra, Imbé	188	67	0.0568	16
6	Barreirinho, Coetinho, Figueira	253	95	0.0806	23
7	Bahia de Campo	257	74	0.0628	18
8	Agrovila, São Benedito	184	66	0.0560	16
9	Agroana	372	178	0. 1510	44
10	Bandeira, Minadouro	248	82	0.0696	20
11	Carretão, Deus Ajuda, Sangradouro, Pesqueiro, Varzearia	216	77	0.0653	19
12	Chafariz, Ramos, Sete Porcos, Urubamba	208	67	0.0568	16
13	Céu Azul, Capão Verde, Morro Cortado, Passagem de Carro, Varal	157	65	0.0551	16

	Total	3,652	1,179	1.0000	290

ID = identification of the microarea.

**Table 2:** Distribution of the 13 communities of Nossa Senhora Aparecida do Chumbo District, Poconé, Mato Grosso, Brazil.

ID	Comunity	Population	Number of individuals^a^	Sample fraction	Sample size	*N*	Plant citations
1	Chumbo	946	216	0.1832	52	50	827
2	Canto do Agostinho, Santa Helena, Os Cagados, Várzea bonita	179	52	0.0441	15	10	131
3	Furnas II, Salobra, Zé Alves	165	59	0.050	15	10	99
4	Campina II, Furnas I, Mundo Novo, Rodeio	279	81	0.0687	20	11	179
5	Campina de Pedra, Imbé	188	67	0.0568	16	12	173
6	Barreirinho, Coetinho, Figueira	253	95	0.0806	23	23	213
7	Bahia de Campo	257	74	0.0628	18	13	461
8	Agrovila, São Benedito	184	66	0.056	16	16	141
9	Agroana	372	178	0.151	44	38	349
10	Bandeira, Minadouro	248	82	0.0696	20	22	171
11	Carretão, Deus Ajuda, Sangradouro, Pesqueiro, Varzearia	216	77	0.0653	19	23	180
12	Chafariz, Ramos, Sete Porcos, Urubamba	208	67	0.0568	16	16	200
13	Céu Azul, Capão Verde, Morro Cortado, Passagem de Carro, Varal	157	65	0.0551	16	18	165
	*N*	3,652	1,179	1.000	290	262	3,289

ID: Identification of the microarea; *N*: Sample size; ^a^Informants with age ≥ 40 years and period of residing ≥ 5 years.

**Table 3:** Relation of the relative importance of the plant species mentioned by informants of Nossa Senhora Aparecida do Chumbo District, Poconé, Mato Grosso, Brazil.

Family/species	Vernacular name	Application	Preparation (administration)	Uses listed	NCS	NP	RI
1. ACANTHACEAE							
1.1. *Justicia pectoralis* Jacq.	Anador	pain, fever, laxative, and muscle relaxant	Infusion (I)	36	2	3	0.40
2. ADOXACEAE							
2.1.* Sambucus australis* Cham. & Schltdl.	Sabugueiro	Fever and measles	Infusion (I, E)	24	2	2	0.33
3. ALISMATACEAE							
3.1.* Echinodorus macrophyllus* (Kuntze.) Micheli	Chapéu-de- couro	blood cleanser, stomach, rheumatism, and kidneys		43	4	4	0.67
4. AMARYLLIDACEAE							
4.1. *Allium cepa *L.	Cebola	wound healing	Infusion (I)	1	1	1	0.17
4.2.* Allium fistulosum* L.	Cebolinha	Flu	Infusion (I)	1	1	1	0.17
4.3.* Allium sativum* L.	Alho	hypertension	Infusion (I)	7	1	1	0.17
5. AMARANTHACEAE							
5.1. *Alternanthera brasiliana* (L.) Kuntze	Terramicina	wound healing, itching, diabetes, pain, bone fractures, throat, flu, inflammation uterine, and relaxative muscular	Infusion (I, E)	41	6	9	1.20
5.2.* Alternanthera dentata*(Moench) Stuchlik ex R.E. Fr.	Ampicilina	wound healing and kidneys	Infusion (I, E)	7	2	2	0.33
5.3. *Alternanthera ficoide* (L.) P. Beauv.	Doril	muscular relaxative	Infusion (I, E)	3	1	1	0.17
5.4*. Amaranthus *aff.* viridis* L.	Caruru-de-porco	wound healing, pain, and kidneys	Infusion (I)	4	3	3	0.50
5.5. *Beta vulgaris* L.	Beterraba	anemia	Infusion (I)	1	1	1	0.17
5.6. *Celosia argentea* L.	Crista-de-galo	kidneys		5	3	3	0.50
5.7. *Chenopodium ambrosioides* L.	Erva-de-santa-maria	wound healing, heart, diabetes, bone fractures, flu, kidneys, cough, and worms	Infusion (I, E)	102	7	8	1.23
5.8.* Pfaffia glomerata* (Spreng.) Pedersen	Ginseng-brasileiro	Obesity	Infusion (I)	2	1	1	0.17
6. ANACARDIACEAE							
6.1. *Anacardium humile* A. St.– Hil.	Cajuzinho-do-campo	diabetes, dysentery, and hepatitis	Infusion (I, E)	5	3	3	0.50
6.2. *Anacardium occidentale *L.	Cajueiro	abortive, wound healing, cholesterol, teeth, blood cleanser, diabetes, diarrhea, dysentery, and pain	Infusion (I, E)	30	6	9	1.20
6.3. *Astronium fraxinifolium* Schott ex Spreng	Gonçaleiro	flu, hemorrhoids, and cough	Infusion and maceration (I, E)	8	3	3	0.50
6.4. *Mangifera indica* L.	Mangueira	Bronchitis, flu, and cough	Infusion and maceration (I, E)	11	2	3	0.40
6.5. *Myracrodruon urundeuva *(Allemão) Engl.	Aroeira	anemia, bladder bronchitis cancer, wound healing, blood cleanser, bone fractures, hernia, uterine inflammation, muscular relaxative, and cough	Infusion, maceration, and decoction (I, E)	84	7	11	1.43
6.6. *Spondias dulcis* Parkinson	Caja-manga	scabies	Infusion (I, E)	2	1	1	0.17
6.7. *Spondias purpurea* L.	Seriguela	wound healing and hepatitis	Infusion (I, E)	2	2	2	0.33
7. ANNONACEAE							
7.1. *Annona cordifolia* Poepp. ex Maas & Westra	Araticum-abelha	Diabetes and bone fractures	Infusion and decoction (I, E)	3	2	2	0.33
7.2. *Annona crassiflora* Mart.	Graviola	diabetes	Infusion (I, E)	11	1	1	0.17
7.3*. Duguetia furfuracea* (A. St.- Hil.) Saff.	Beladona-do-cerrado	pain	Infusion (I, E)	1	1	1	0.17
8. APIACEAE							
8.1. *Coriandrum sativum* L.	Coentro	flu	Infusion (I)	1	1	1	0.17
8.2.* Eryngium* aff. *pristis* Cham. & Schltdl.	Lingua-de-tucano	Tooth and muscular relaxative	Infusion (I)	3	2	2	0.33
8.3.* Petroselinum crispum* ((Mill) Fuss	Salsinha	flu	Infusion (I)	1	1	1	0.17
8.4. *Pimpinella anisum* L.	Erva-doce	pain soothing, constipation, and kidneys	Infusion (I, E)	12	3	3	0.50
9. APOCYNACEAE							
9.1. *Aspidosperma polyneuron *(Müll.) Arg.	Péroba	Stomach and laxative	Infusion and decoction (I, E)	5	1	2	0.23
9.2. *Aspidosperma tomentosum* Mart.	Guatambu	gastritis	Infusion (I)	4	1	1	0.17
9.3.* Catharanthus roseus* (L.) G. Don	Boa-noite	mumps fever and kidneys	Infusion (I)	8	3	3	0.50
9.4. *Geissospermum laeve* (Vell.) Miers	Pau-tenente	Diabetes and pain	Infusion (I)	6	2	2	0.33
9.5. *Hancornia speciosa* var. *gardneri (*A. DC.) Müll. Arg.	Mangava-mansa	itching, diarrhea, and stomach	Decoction and maceration (I, E)	8	3	3	0.50
9.6. *Himatanthus obovatus *(Müll. Arg.) Woodson	Angélica	anemia, wound healing, cholesterol, blood cleanser, pain, nose bleeding, hypertension, uterine inflammation, labyrinthitis, pneumonia, relaxative muscular, worms, and vitiligo	Maceration (I)	45	10	13	1.87
9.7.* Macrosiphonia longiflora* (Desf.) Müll. Arg.	Velame-do-campo	hearth, blood cleanser, stroke, diuretic, pain, throat, muscular relaxative, and vitiligo	Decoction (I)	5	6	8	1.13
9.8. *Macrosiphonia velame* (A. St.-Hil.) Müll. Arg.	Velame-branco	flu	Decoction (I)	73	1	1	0.17
10. ARACEAE							
10.1* Dieffenbachia picta* Schott	Comigo-ninguém-pode	pain	Maceration (E)	2	1	1	0.17
10.2*. Dracontium* sp.	Jararaquinha	snakebite	Infusion (I)	10	1	1	0.17
11. ARECACEAE							
11.1. *Acrocomia aculeata* Lodd. ex. Mart.	Bocaiuveira	heart, hepatitis, hypertension, and kidneys	Decoction and syrup (I)	20	4	4	0.67
11.2.* Cocos nucifera* L.	Cocô-da-bahia	kidneys	Maceration (I)	2	1	1	0.17
11.3. *Orbignya phalerata* Mart.	Babaçu	inflammation	Decoction (I)	8	1	1	0.17
11.4.* Syagrus oleracea* (Mart.) Becc.	Guariroba	kidneys	Maceration (I)	2	1	1	0.17
12. ARISTOLOCHIACEAE							
12.1. *Aristolochia cymbifera* Mart & Zucc.	Cipó-de-mil-homem	dengue, blood cleanser, stomach, kidneys, and digestive	Infusion (I)	11	4	5	0.73
12.2.* Aristolochia esperanzae* Kuntze	Papo-de-peru	wound healing	Infusion (I)	3	1	1	0.17
13. ASTERACEAE							
13.1. *Acanthospermum australe* (Loefl.). Kuntze	Carrapicho, beijo-de-boi	colic, kidneys, and runny cough	Infusion (I)	31	2	3	0.40
13.2.* Acanthospermum hispidum* DC.	Chifre-de-garrotinho	Gonorrhea and kidneys	Infusion (I)	5	2	3	0.40
13.3. *Achillea millefolium* L.	Dipirona, Novalgina,	pain, flu, and muscular relaxative	Infusion (I)	13	3	4	0.57
13.4. *Achyrocline satureioides *(Lam.) DC.	Macela-do-campo	diarrhea, pain, stomach, gastritis, flu, and hypertension	Infusion (I)	13	5	6	0.90
13.5. *Ageratum conyzoides* L.	Mentrasto	pain, labor pain, stomach, swelling in pregnant woman, rheumatism, and cough	Infusion (I)	18	5	6	0.90
13.6. *Artemisia vulgaris* L.	Artemisia	insomnia	Infusion (I)	3	1	1	0.17
13.7. *Artemisia absinthium* L.	Losna, nor-vômica	pain, stomach, liver, hernia, and muscular relaxative	Infusion (I)	39	4	5	0.73
13.8. *Baccharis trimera* (Less.) DC.	Carqueja	cancer, cholesterol, diabetes, diuretic, stomach, flu, and obesity	Infusion (I)	31	5	7	0.97
13.9. *Bidens pilosa* L.	Picão-preto	hepatitis, enteric, and kidneys	Infusion (I, E)	20	3	3	0.50
13.10. *Brickellia brasiliensis* (Spreng.) B.L. Rob.	Arnica-do-campo	wound healing, uterine inflammation, and kidneys	Infusion (I)	13	2	3	0.40
13.11. *Calendula officinalis* L.	Calêndula	anxiety	Infusion (I)	6	1	1	0.17
13.12. *Centratherum* aff. *punctatum* Cass.	Perpétua-roxa	muscular relaxative, and hearth	Infusion (I)	3	2	2	0.33
13.13. *Chamomilla recutita* (L.) Rauschert.	Camomila	soothing colic, pain, stomach, fever, and flu	Infusion (I)	78	5	6	0.90
13.14*. Chaptalia integerrima* (Vell.) Burkart	Lingua-de-vaca	worms	Infusion (I)	6	1	1	0.17
13.15. *Chromolaena odorata* (L.) R.M. King & H. Rob	Cruzeirinho	colic, pain, bone fractures, pain, bone fractures, and kidneys	Infusion (I)	7	3	4	0.57
13.16. *Conyza bonariensis* (L.) Cronquist	Voadeira	cancer itching, blood cleanser, leukemia, and worms	Infusion (I)	15	4	5	0.73
13.17.* Elephantopus mollis* Kunth	Sussuaiá	blood cleanser, pain, and uterine inflammation	Infusion (I)	11	2	3	0.40
13.18. *Emilia fosbergii* Nicolson	Serralha	conjunctivitis	Infusion (I)	6	1	1	0.17
13.19. *Eremanthus exsuccus* (DC.) Baker	Bácimo-do-campo	wound healing, stomach, bone fractures, and skin	Infusion and maceration (I, E)	11	3	4	0.57
13.20. *Eupatorium odoratum* L.	Arnicão	wound healing, muscular relaxative, and kidneys	Infusion (I, E)	10	3	3	0.50
13.21. *Mikania glomerata* Spreng.	Guaco	bronchitis cough	Infusion (I)	14	2	2	0.33
13.22. *Mikania hirsutissima* DC.	Cipó-cabeludo	diabetes	Infusion (I)	10	1	1	0.17
13.23. *Pectis jangadensis* S. Moore	Erva-do-carregador	blood cleanser and diabetes	Infusion (I)	4	2	2	0.33
13.24.* Porophyllum ruderale* (Jacq.) Cass.	Picão-branco	Hepatitis and kidneys	Infusion (I)	11	2	2	0.33
13.25. *Solidago microglossa* DC.	Arnica-brasileira	wound healing, blood cleanser, pain, bone fractures, hypertension, uterine inflammation, muscular relaxative, kidneys, worms, pain, stomach, hypertension, pneumonia, constipation, and relaxative muscular	Infusion (I, E)	82	8	15	1.80
13.26. *Spilanthes acmella* (L.) Murray	Jambú	liver	Infusion (I)	5	1	1	0.17
13.27.* Tagetes minuta* L.	Cravo-de-defunto	Dengue and flu	Infusion (I)	3	2	2	0.33
13.28.* Taraxacum officinale* L.	Dente-de-leão	blood cleanser	Infusion (I)	18	1	1	0.17
13.29. *Tithonia diversifolia* (Hemsl.) A. Gray	Flor-da-amazônia	alcoholism, stomach, kidney, and constipation	Infusion (I)	16	3	3	0.50
13.30.*Vernonia condensata* Baker	Figatil-caferana	cancer stomach and liver	Infusion (I)	48	2	3	0.40
13.31. *Vernonia scabra* Pers.	Assa-peixe	bronchitis blood cleanser, fever, flu, pneumonia, cold, and cough	Infusion and syrup (I)	38	2	7	0.67
13.32. *Zinnia elegans* Jacq.	Jacinta	pain	Infusion (I)	1	1	1	0.17
14. BERBERIDACEAE							
14.1. *Berberis laurina* Billb.	Raiz-de-são-joão	blood cleanser and diarrhea	Decoction and bottle (I, E)	6	2	2	0.33
15. BIGNONIACEAE							
15.1*. Anemopaegma arvense* (Vell.) Stellfeld & J.F. Souza	Verga-teso, Alecrim-do-campo, Catuaba	anxiety soothing kidneys	Decoction and bottle (I, E)	13	2	3	0.40
15.2. *Arrabidaea chica* (Humb. & Bonpl.) B. Verl.	Crajirú	wound healing and blood cleanser	Infusion (I)	6	2	2	0.33
15.3. *Cybistax antisyphilitica* (Mart.) Mart.	Pé-de-anta	fever, flu, relaxative muscular, and worms	Infusion (I)	13	4	4	0.67
15.4. *Jacaranda caroba* (Vell.) A. DC.	Caroba	wound healing	Decoction and bottle (I, E)	3	1	1	0.17
15.5. *Jacaranda decurrens* Cham.	Carobinha	allergy cancer wound healing, blood cleanser, diabetes, leprosy, hemorragia no nariz, inflammation uterina, and kidneys	Decoction and bottle (I, E)	94	8	9	1.40
15.6. *Tabebuia aurea* (Silva Manso) B. & H. f. ex S. Moore	Ipê-amarelo	worms	Decoction and bottle (I)	2	1	1	0.17
15.7. *Tabebuia caraiba* (Mart.) Bureau	Para-tudo	prostate cancer anemia, bronchitis cancer blood cleanser, diarrhea, pain, stomach, cough, and worms	Decoction and bottle (I, E)	67	6	10	1.27
15.8. *Tabebuia impetiginosa* (Mart. ex DC.) Standl.	Ipê-roxo	prostate cancer cough	Decoction and bottle (I)	8	2	2	0.33
15.9. *Tabebuia serratifolia* Nicholson	Piúva	prostate cancer	Decoction and bottle (I, E)	3	1	1	0.17
15.10. *Zeyhera digitalis* (Vell.) Hochn.	Bolsa-de-pastor	Stomach	Decoction and bottle (I)	9	1	1	0.17
16. BIXACEAE							
16.1. *Bixa orellana* L.	Urucum	cholesterol, stroke, bone fractures, and measles	Infusion (I)	11	4	4	0.67
16.2. *Cochlospermum regium* (Schrank) Pilg.	Algodãozinho-do-campo	blood cleanser, stomach, bone fractures, inflammation uterina, syphilis, vitiligo, gonorrhea, and ringworm	Infusion (I)	37	6	9	1.20
17. BOMBACACEAE							
17.1. *Pseudobombax longiflorum* (Mart. Et Zucc.) Rob.	Embiriçu-do-cerrado	pneumonia, cough, and tuberculosis	Infusion (I)	17	3	3	0.50
17.2. *Eriotheca candolleana* (K. Schum.)	Catuaba	prostate cancer		1	1	1	0.17
18. BORAGINACEAE							
18.1. *Cordia insignis* Cham.	Calção-de-velho	cough	Infusion (I)	5	1	1	0.17
18.2. *Heliotropium filiforme* Lehm.	Sete-sangria	thooth, blood cleanser, hypertension, and tuberculosis	Infusion (I)	43	4	4	0.67
18.3. *Symphytum asperrimum* Donn ex Sims	Confrei	wound healing, heart, throat, and obesity	Infusion (I, E)	10	4	4	0.67
19. BRASSICACEAE							
19.1. *Nasturtium officinale* R. Br.	Agrião	bronchitis	Infusion (I)	2	1	1	0.17
20. BROMELIACEAE							
20.1. *Ananas comosus* (L.) Merr.	Abacaxi	diuretic and cough	Infusion (I)	9	2	2	0.33
20.2. *Bromelia balansae* Mez	Gravatá	cough and bronhitis	Infusion (I)	2	2	2	0.33
21. BURSERACEAE							
21.1. *Commiphora myrrha* (T. Nees) Engl.	Mirra	Menstruation and rheumatism	Infusion (I)	3	2	2	0.33
21.2.* Protium heptaphyllum *(Aubl.) Marchand	Almésica	blood cleanser, stroke, pain, muscular relaxative, rheumatism, and cough		23	3	6	0.70
22. CACTACEAE							
22.1. *Cactus alatus* Sw.	Cacto	Colic and guard delivery	Infusion (I, E)	10	2	2	0.33
22.2. *Opuntia* sp.	Palma	column		2	1	1	0.17
22.3. *Pereskia aculeata *Mill.	Oro-pro-nobis	anemia	Infusion (I)	2	1	1	0.17
23. CAPPARACEAE							
23.1. *Crataeva tapia* L.	Cabaça	cough	Infusion (I)	2	1	1	0.17
23.2. *Cleome affinis DC. *	Mussambé	diarrhea		1	1	1	0.17
24. CARICACEAE							
24.1. *Carica papaya* L.	Mamoeiro	worms, thooth, stomach, hepatitis, muscular relaxative, and cough	Infusion (I)	17	4	6	0.80
25. CARYOCARACEAE							
25.1. *Caryocar brasiliense* A. St.-Hil.	Pequizeiro	diabetes, hypertension, labyrinthitis, and obesity		11	4	4	0.67
26. CELASTRACEAE							
26.1. *Maytenus ilicifolia* Mart.ex Reissek	Espinheira-santa	uric acid, bronchitis diarrhea, stomach, gastritis, flu, and cough	Infusion (I)	8	5	7	0.97
27. CECROPIACEAE							
27.1. *Cecropia pachystachya* Trécul	Embaúba	cholesterol, blood cleanser, diabetes, pain, hypertension, leukemia, pneumonia, kidneys, and cough	Infusion (I)	38	6	9	1.20
28. CLUSIACEAE							
28.1. *Kielmeyera* aff. *grandiflora* (Wawra) Saddi	Pau-santo	anemia		1	1	1	0.17
29. COMBRETACEAE							
29.1. *Terminalia argentea* Mart.	Pau-de-bicho	itching, diabetes, and cough		8	3	3	0.50
29.2. *Terminalia catappa* L.	Sete-copa	conjunctivitis	Infusion (I, E)	2	1	1	0.17
30. COMMELINACEAE							
30.1. *Commelina benghalensis* L.	Capoeraba	hemorrhoids	Infusion (I)	1	1	1	0.17
30.2. *Commelina nudiflora* L.	Erva-de-santa-luzia	wound healing and conjunctivitis	Infusion (I)	3	2	2	0.33
30.3. *Dichorisandra hexandra* (Aubl.) Standl.	Cana-de-macaco	flu, hypertension, and kidneys	Infusion (I)	1	3	3	0.50
31. CONVOLVULACEAE							
31.1.* Cuscuta racemosa* Mart.	Cipó-de-chumbo	pain	Infusion (I)	1	1	1	0.17
31.2*. Ipomoea batatas* (L.) Lam.	Batata-doce	hearth	Infusion (I)	1	1	1	0.17
31.3.* Ipomoea* (Desr.) Roem. & asarifolia Schult	Batatinha-do-brejo	Stomach and worms	Infusion (I)	4	2	2	0.33
32. COSTACEAE							
32.1. *Costus spicatus* (Jacq.) Sw.	Caninha-do-brejo	bladder diuretic, inflammation uterina, muscular relaxative, and kidneys	Infusion (I)	40	3	5	0.63
33. CRASSULACEAE							
33.1. *Kalanchoe pinnata* (Lam.) Pers.	Folha-da-fortuna	allergy, bronchitis blood cleanser, and flu	Infusion and juice (I)	11	2	4	0.47
34. CUCURBITACEAE							
34.1. *Cayaponia tayuya* (Cell.) Cogn.	Raiz-de-bugre	blood cleanser, pain, and hepatitis	Infusion (I)	17	2	3	0.40
34.2. *Citrullus vulgaris* Schrad.	Melância	bladder colic	Infusion (I)	2	1	2	0.23
34.3.* Cucumis anguria* L.	Máxixe	anemia	Infusion (I)	1	1	1	0.17
34.4. *Cucumis sativus* L.	Pepino	hypertension	Maceration (I)	1			
34.5. *Cucurbita maxima* Duchesne ex Lam.	Abóbora	Pain and worms	Infusion (I)	4	2	2	0.33
34.6. *Luffa* sp	Bucha	Anemia and kidneys	Infusion (I)	7	2	2	0.33
34.7. *Momordica charantia* L.	Melão-de-são-caetano	bronchitis dengue, stomach, fever, flu, hepatitis, swelling in pregnant woman, malaria, muscular relaxative, and worms	Infusion (I)	50	6	10	1.27
34.8. *Siolmatra brasiliensis* (Cogn.) Baill.	Taiuá	Ulcer	Infusion (I)	6	1	1	0.17
35. CYPERACEAE							
35.1. *Bulbostylis capillaris* (L.) C.B. Clarke	Barba-de-bode	diuretic, stomach, kidneys, and worms	Infusion (I)	12	3	4	0.57
35.2.* Cyperus rotundus* L.	Tiririca	Pain	Infusion (I)	1	1	1	0.17
36. DILLENIACEAE							
36.1. *Curatella americana* L.	Lixeira	wound healing, colic, diarrhea, flu, kidneys, and cough	Infusion (I, E)	24	5	6	0.90
36.2. *Davilla elliptica* A. St.-Hil.	Lixeira-de-cipó	kidneys		3	1	1	0.17
36.3. *Davilla nitida* (Vahl.) Kubitzki	Lixeirinha	delivery help, liver, hernia, and kidneys	Infusion (I)	10	3	4	0.57
37. DIOSCOREACEAE							
37.1. *Dioscorea* sp.	Cará-do-cerrado	boil	Infusion (I)	25	1	1	0.17
37.2. *Dioscorea trifida* L	Cará	blood cleanser	Infusion (I)	6	1	1	0.17
38. EBENACEAE							
38.1. *Diospyros hispida* A. DC.	Olho-de-boi	Pain and leprosy	Infusion (I)	5	2	2	0.33
39. EQUISETACAE							
39.1. *Equisetum arvense* L.	Cavalinha	gastritis and kidneys	Infusion (I)	8	2	2	0.33
40. ERYTHROXYLACEAE							
40.1 *Erythroxylum* aff. *Daphnites* Mart.	Vasoura-de-bruxa	syphilis	Infusion (I)	1	1	1	0.17
41. EUPHORBIACEAE							
41.1. *Croton antisyphiliticus* Mart.	Curraleira	Hypertension and uterine inflammation	Infusion (I)	6	2	2	0.33
41.2. *Croton* sp.	Curraleira-branca	uterine inflammation	Infusion (I)	3	1	1	0.17
41.3. *Croton urucurana* Baill.	Sangra-d'água	cancer prostate cancer healing, diabetes, stomach, gastritis, uterine inflammation, kidneys, and ulcer	Maceration (I)	37	5	9	1.10
41.4. *Euphorbia* aff. *Thymifolia* L.	Trinca-pedra	kidneys	Infusion (I)	3	1	1	0.17
41.5. *Euphorbia prostrata* Aiton	Fura-pedra	kidneys	Infusion (I)	4	1	1	0.17
41.6. *Euphorbia tirucalli* L	Aveloz	cancer uterine inflammation	Maceration (I)	3	2	2	0.33
41.7. *Jatropha* sp.	Capa-rosa	diabetes	Infusion (I)	10	1	1	0.17
41.8. *Jatropha elliptica* (Poh) Oken	Purga-de-lagarto	allergy	Infusion (I)	38	1	1	0.17
41.9. *Jatropha* aff. *Gossypiifolia* L.	Pinhão-roxo	wound healing, prostrate cancer, itching, blood cleanser, stroke, snakebite, syphilis, worms, and vitiligo	Maceration(I, E)	7	6	10	1.27
41.10. *Jatropha urens* L.	Cansansão	diabetes	Maceration (I, E)	6	1	1	0.17
41.11. *Manihot esculenta* Crantz	Mandioca-braba	itching	Maceration (I, E)	2	1	1	0.17
41.12. *Manihot utilissima* Pohl.	Mandioca	itching	Maceration (I, E)	7	1	1	0.17
41.13. *Ricinus communis* L.	Mamona	wound healing and blood cleanser	Maceration (I, E)	8	2	2	0.33
41.14. *Synadenium grantii* Hook. f.	Cancerosa	gastritis, prostate cancer stomach, and pneumonia	Maceration (I, E)	12	3	4	0.57
42. FABACEAE							
42.1. *Acosmium dasycarpum* (Volgel) Yakovlev	Cinco-folha	column, blood cleanser, pain, and kidneys	Infusion (I)	19	2	4	0.47
42.2. *Acosmium subelegans* (Mohlenbr.) Yakovlev	Quina-gensiana	wound healing, blood cleanser, pain, liver, uterine inflammation, delivery relapse, and kidneys	Decoction (I)	16	5	7	0.97
42.3. *Albizia niopoides* (Spr. ex Benth.) Burkart.	Angico-branco	bronhitis	Decoction (I)	1	1	1	0.17
42.4. *Amburana cearensis* (Allemão) A. C. Sm.	Imburana	cough	Decoction (I)	13	1	1	0.17
42.5. *Anadenanthera colubrina* (Vell.) Brenan	Angico	asthma, wound healing, expectorant, uterine inflammation, pneumonia, and cough	Decoction (I)	12	5	6	0.90
42.6. *Andira anthelminthica* Benth.	Angelim	diabetes	Decoction (I)	3	1	1	0.17
42.7. *Bauhinia variegata* L.	Unha-de-boi	kidneys	Decoction (I)	4	1	1	0.17
42.8. *Bauhinia ungulata* L.	Pata-de-vaca	diabetes	Infusion (I)	11	1	1	0.17
42.9. *Bauhinia glabra* Jacq.	Cipó-tripa-de-galinha	diarrhea, dysentery, and pain	Infusion (I)	7	3	3	0.50
42.10. *Bauhinia rubiginosa* Bong.	Tripa-de-galinha	kidneys	Infusion (I)	2	1	1	0.17
42.11. *Bauhinia rufa* (Bong.) Steud.	Pata-de-boi	diabetes	Infusion (I)	1	1	1	0.17
42.12. *Bowdichia virgilioides* Kunth	Sucupira	blood cleanser, paom, stomach, nose bleeding, cough, and worms	Bottle (I)	20	4	6	0.80
42.13. *Caesalpinia ferrea* Mart.	Jucá	wound healing, stomach, bone fractures, and inflammation of uterine	Maceration (I, E)	15	3	4	0.57
42.14. *Cajanus bicolor* DC.	Feijão-andu	diarrhea, stomach and worms	Infusion (I)	8	2	3	0.40
42.15. *Cassia desvauxii* Collad.	Sene	constipation, pain, fever, uterine inflammation, and labyrinthitis	Infusion (I)	18	4	5	0.73
42.16. *Chamaecrista desvauxii* (Collad.) Killip	Sene-do-campo	constipation, blood cleanser, pain, and fever	Infusion (I)	10	2	4	0.47
42.17. *Copaifera *sp.	Pau-d'óleo	wound healing, kidneys, ulcer	Infusion (I)	8	3	3	0.50
42.18.* Copaifera langsdorffii* var. *glabra *(Vogel) Benth.	Copaiba	bronchitis prostate cancer stroke, pain, throat, and tuberculosis	Maceration and syrup (I)	13	5	6	0.90
42.19. *Copaifera marginata* Benth.	Guaranazinho	ulcer	Infusion (I)	4	1	1	0.17
42.20. *Desmodium incanum* DC.	Carrapicho	bladder itching, diarrhea, pain, hepatitis, and kidneys	Infusion (I)	18	5	6	0.90
42.21. *Dimorphandra mollis* Benth.	Fava-de-santo-inácio	bronchitis wound healing, pain, flu, hypertension, pneumonia, rheumatism, cough, and worms	Infusion (I)	21	6	9	1.20
42.22. *Dioclea latifolia* Benth.	Fruta-olho-de-boi	stroke	Infusion (I)	3	1	1	0.17
42.23. *Dioclea violacea* Mart. Zucc.	Coronha-de-boi	osteoporosis	Infusion (I)	6	2	2	0.33
		stroke					
42.24. *Dipteryx alata* Vogel	Cumbarú	bronchitis cicartrizante, diarrhea, dysentery, pain, throat, flu, snakebite, and cough	Infusion (I)	43	4	9	1.00
42.25. *Galactia glaucescens* Kunth	Três-folhas	column, pain, bone fractures, and kidneys	Infusion (I)	8	4	4	0.67
42.26. *Hymenaea courbaril* L.	Jatobá-mirim	bladder bronchitis flu, pneumonia, and cough	Syrup and decoction (I)	36	3	5	0.63
42.27. *Hymenaea stigonocarpa* Mart. ex Hayne	Jatoba-do-cerrado	bronchitis prostate cancer pain, fertilizer, flu, and cough	Syrup and decoction (I)	31	5	6	0.90
42.28. *Indigofera suffruticosa* Mill.	Anil	ulcer	Infusion (I)	2	1	1	0.17
42.29. *Inga vera* Willd.	Ingá	Laxative and kidneys	Infusion (I)	5	2	2	0.33
42.30. *Machaerium hirtum* (Vell.) Stellfeld	Espinheira-santa-nativa	ulcer	Infusion (I)	2	1	1	0.17
42.31.* Melilotus officinalis* (L) Pall.	Trevo-cheiroso	bone fractures and thyroid	Infusion (I)	5	2	2	0.33
42.32.* Mimosa debilis* var. vestita (Benth.) Barneby	Dorme-dorme	soothing	Infusion (I)	2	1	1	0.17
42.33. *Mucuna pruriens* (L.) DC.	Macuna	stroke	Infusion (I)	2	1	1	0.17
42.34. *Peltophorum dubium* (Spreng.) Taub.	Cana-fistula	gastritis	Infusion (I)	5	1	1	0.17
42.35. *Platycyamus regnellii* Benth.	Pau-porrete	anemia	Infusion (I)	1	1	1	0.17
42.36. *Pterodon pubescens* (Benth.) Benth.	Sucupira-branca	worms, pain, and stomach	SYRope, decoction and maceration (I)	2	3	3	0.50
42.37. *Senna alata* (L.) Roxb.	Mata-pasto	throat, worms, and vitiligo	Infusion (I)	6	3	3	0.50
42.38. *Senna occidentalis* (L.) Link	Fedegoso	blood cleanser, pain, flu, cough, and worms	Infusion (I)	42	3	5	0.63
42.39. *Stryphnodendron obovatum *Benth.	Barbatimão 1	wound healing	Syrup and decoction (I, E)	57	1	1	0.17
42.40. *Stryphnodendron adstringens* (Mart.) Coville	Barbatimão 2	bladder bronchitis, colic, stomach, bone fractures, uterine inflammation, relaxative muscular, and ulcer	Syrup and decoction (I, E)	15	4	9	1.00
42.41.* Tamarindus indica* L.	Tamarindo	anxiety pain, thooth, laxative, osteoporosis, syphilis, and worms	Maceration and juice (I)	30	6	7	1.07
43. FLACOURTIACEAE							
43.1. *Casearia silvestris* Sw.	Guaçatonga	Epilepsy and kidneys	Infusion (I)	3	2	2	0.33
44. GINKGOACEAE							
44.1. *Ginkgo biloba* L.	Ginco-biloba	vertebral	Infusion (I)	1	1	1	0.17
45. HERRERIACEAE							
45.1.* Herreria salsaparilha* Mart.	Salsaparilha	column, blood cleanser, muscular relaxative, and kidneys	Infusion (I)	12	3	4	0.57
46. HIPPOCRATEACEAE							
46.1. *Salacia* aff. *elliptica* (Mart. ex Schult.) G. Don	Saputa-do-brejo	pain	Infusion (I)	6	1	1	0.17
47. IRIDACEAE							
47.1. *Eleutherine bulbosa* (Mill.) Urb.	Palmeirinha	pain, hemorrhoids, cough, and blood cleanser	Infusion (I)	11	2	4	0.47
48. LAMIACEAE							
48.1. *Hyptis* cf. *hirsuta* Kunth	Hortelã-do-campo	diabetes, stomach, flu, cough, and worms	Infusion (I)	23	5	5	0.83
48.2. *Hyptis paludosa* St.-Hil.ex Benht.	Alevante	cold	Infusion (I)	4	1	1	0.17
48.3. *Hyptis* sp.	Hortelã-bravo	Diabetes and cough	Infusion (I)	6	2	2	0.33
48.4. *Hyptis suaveolens* (L.) Poit.	Tapera-velha	pain, stomach, flu, constipation, kidneys, and worms	Infusion (I)	42	5	6	0.90
48.5. *Leonotis nepetifolia* (L.) R. Br.	Cordão-de-são-francisco	column, hearth, blood cleanser, stomach, fever, gastritis, flu, hypertension, labyrinthitis, muscular relaxative, and kidneys	Infusion (I)	38	7	11	1.43
48.6. *Marsypianthes chamaedrys* (Vahl) Kuntze	Alfavaca/Hortelã-do-mato	flu, hypertension, and cough	Infusion (I)	8	3	3	0.50
48.7. *Melissa officinalis* L	Melissa	soothing	Infusion (I)	2	1	1	0.17
48.8. *Mentha crispa* L.	Hortelã-folha-miuda	anemia, liver, cough, and worms	Infusion (I)	16	4	4	0.67
48.9.* Mentha pulegium* L.	Poejo	bronchitis soothing fever, flu, cold, and cough	Infusion (I)	59	3	6	0.70
48.10. *Mentha spicata* L.	Hortelã-vicki	bronchitis flu, wound healing, stomach, and worms	Infusion (I)	24	4	5	0.73
48.11. *Mentha *x* piperita* L.	Hortelã-pimenta	bronchitis flu, cough and worms	Infusion (I)	42	3	4	0.57
48.12. *Mentha x villosa* Huds.	Hortelã-rasteira	stomach, flu, cold, and worms	Infusion (I)	86	3	4	0.57
48.13. *Ocimum kilimandscharicum* Baker ex Gürke	Alfavacaquinha	flu	Infusion (I)	2	1	1	0.17
48.14. *Ocimum minimum* L.	Manjericão	kidneys, sinusitis, and worms	Infusion (I)	7	3	3	0.50
48.15. *Origanum majorana* L.	Manjerona	heart	Infusion (I)	4	1	1	0.17
48.16. *Origanum vulgare *L.	Orégano	cough	Infusion (I)	1	1	1	0.17
48.17. *Plectranthus amboinicus* (Lour.) Spreng.	Hortelã-da-folha-gorda	bronchitis flu, uterine inflammation, and cough	Infusion and syrup (I)	7	3	4	0.57
48.18. *Plectranthus barbatus* Andrews	Boldo-brasileiro	pain, stomach, liver, and malaise	Maceration (I)	99	2	4	0.47
48.19. *Plectranthus neochilus* Schltr.	Boldinho	stomach	Maceration (I)	1	1	1	0.17
48.20.* Rosmarinus officinalis* L.	Alecrim	anxiety soothing hearth, pain, hypertension, insomnia, labyrinthitis, sluggishness memory, tachycardia, and vitiligo	Infusion and maceration (I)	31	6	10	1.27
49. LAURACEAE							
49.1. *Cinnamomum camphora* (L.) Nees & Eberm.	Cânfora	pain	Infusion and maceration (I)	1	1	1	0.17
49.2. *Cinnamomum zeylanicum* Breyne	Canela-da-india	aphrodisiac, tonic, obesity, and cough	Infusion (I)	11	3	4	0.57
49.3.* Persea americana* Mill.	Abacateiro	diuretic, hypertension, and kidneys	Infusion and maceration (I)	31	3	3	0.50
50. LECYTHIDACEAE							
50.1. *Cariniana rubra* Gardner ex Miers	Jequitibá	bladder wound healing, colic, pain, uterine inflammation, rheumatism, cough, and ulcer	Infusion and maceration (I)	49	5	8	1.03
51. LOGANIACEAE							
51.1. *Strychnos pseudoquina* A. St.-Hil.	Quina	anemia, wound healing, cholesterol, blood cleanser, pain, stomach, bone fractures, flu, uterine inflammation, pneumonia, muscle relaxant, cough, ulcer, and worms	Decoction and maceration (I, E)	107	8	14	1.73
52. LORANTHACEAE							
52.1.* Psittacanthus calyculatus* (D.C.) G. Don	Erva-de-passarinho	stroke, pain, flu, and pneumonia	Infusion and maceration (I)	14	3	4	0.57
53. LYTHRACEAE							
53.1. *Adenaria floribunda *Kunth	Veludo-vermelho	kidneys		3	1	1	0.17
53.2. *Lafoensia pacari *A. St.-Hil.	Mangava-braba	wound healing, diarrhea, pain, stomach, gastritis, kidneys, and ulcer	Decoction and maceration (I, E)	73	5	7	0.97
54. MALPIGHIACEAE							
54.1. *Byrsonima orbignyana* A. Juss.	Angiquinho	wound healing	Decoction and maceration (I)	2	1	1	0.17
54.2. *Byrsonima* sp.	Semaneira	pain	Infusion (I)	1	1	1	0.17
54.3.* Byrsonima verbascifolia* (L.) DC.	Murici-do-cerrado	column	Infusion (I)	3	2	2	0.33
		uterine inflammation					
54.4. *Camarea ericoides* A. St.-Hil.	Arniquinha	wound healing	Infusion (I)	11	1	1	0.17
54.5. *Galphimia brasiliensis* (L.) A. Juss.	Mercúrio-do-campo	wound healing, itching, thooth, and bone fractures	Infusion (I)	7	3	4	0.57
54.6. *Heteropterys aphrodisiaca* O. Mach.	Nó-de-cachorro	brain, wound healing, blood cleanser, impotence, muscular relaxative, and rheumatism	Decoction (I)	23	5	6	0.90
54.7. *Malpighia emarginata* DC.	Cereja	wound healing	Infusion (I)	5	1	1	0.17
54.8. *Malpighia glabra* L.	Aceroleira	bronchitis dengue, stomach, fever, and flu	Infusion (I)	24	4	5	0.73
55. MALVACEAE							
55.1.* Brosimum gaudichaudii* Trécul	Mama-cadela	stomach	Infusion (I)	13	1	1	0.17
55.2. *Gossypium barbadense* L.	Algodão-de-quintal	blood cleanser, stomach, vitiligo, inflammation, and gonorrhea	Infusion (I)	47	5	5	0.83
55.3.* Guazuma ulmifolia* var. *tomentosa* (Kunth) K. Schum.	Chico-magro	diarrhea, kidneys, bronchitis wound healing	Infusion and decoction (I)	10	4	4	0.67
55.4.* Hibiscus pernambucensis* Bertol.	Algodão-do-brejo	wound healing, colic, flu, and uterine inflammation	Infusion (I)	2	3	4	0.57
55.5. *Hibiscus rosa-sinensis* L.	Primavera	pain	Infusion (I)	2	1	1	0.17
55.6. *Hibiscus sabdariffa* L.	Quiabo-de-angola, Hibisco	anxiety hearth, flu, tachycardia, kidneys, colic, runny, diarrhea, pain, uterine inflammation, labyrinthitis, snakebite, and pneumonia	Infusion (I)	18	10	13	1.87
55.7. *Helicteres sacarolha* A. St.-Hil.	Semente-de-macaco	Hypertension and ulcer	Infusion (I)	2	2	2	0.33
55.8*. Malva sylvestris* L.	Malva-branca	wound healing, conjunctivitis, runny, blood cleanser, diuretic, boil, uterine inflammation, and rheumatism	Infusion (I)	31	7	8	1.23
55.9. *Malvastrum corchorifolium* (Desr.) Britton ex Small	Malva	tonsillitis wound healing, pain, and uterine inflammation	Infusion (I)	13	4	4	0.67
55.10. *Sida rhombifolia *L.	Guaxuma	obesity	Infusion (I)	5	1	1	0.17
56. MELASTOMATACEAE							
56.1.* Leandra purpurascens* (DC.) Cogn.	Pixirica	rheumatism	Infusion (I)	1	1	1	0.17
56.2. *Tibouchina clavata* (Pers.) Wurdack	Cibalena	pain	Infusion (I)	3	1	1	0.17
56.3. *Tibouchina urvilleana* (DC.) Cogn.	Buscopam-de-casa	stomach	Infusion (I)	1	1	1	0.17
57. MELIACEAE							
57.1. *Azadirachta indica* A. Juss.	Neem	diabetes	Infusion and decoction (I, E)	1	1	1	0.17
57.2. *Cedrela odorata* L.	Cedro	wound healing	Infusion (I)	3	1	1	0.17
58. MENISPERMACEAE							
58.1. *Cissampelos* sp.	Orelha-de-onça	Column and kidneys	Infusion (I)	3	2	2	0.33
59. MORACEAE							
59.1. *Artocarpus integrifolia* L.f.	Jaca	diuretic	Infusion (I)	1	1	1	0.17
59.2. *Chlorophora tinctoria* (L.) Gaudich. ex Benth.	Taiúva	osteoporosis and muscular relaxative	Infusion (I)	2	2	2	0.33
59.3. *Dorstenia brasiliensis* Lam.	Carapiá	wound healing, colic, thooth, blood cleanser, dysentery, pain, flu, laxative, menstruation, pneumonia, relapse delivery, and kidneys	Infusion (I)	41	7	12	0.50
59.4. *Ficus brasiliensis* Link.	Figo	gastritis	Infusion (I)	4	1	1	0.17
59.5. *Ficus pertusa* L. f.	Figueirinha	stomach	Infusion (I)	5	1	1	0.17
60. MUSACEAE							
60.1. *Musa x paradisiaca* L.	Bananeira-de-umbigo	bronchitis anemia and pain	Infusion and syrup (I)	9	3	3	0.50
61. MYRTACEAE							
61.1. *Eucalyptus citriodora* Hook.	Eucálipto	bronchitis diabetes, fever, flu, sinusitis, and cough	Infusion and syrup (I)	22	3	6	0.70
61.2. *Eugenia pitanga* (O. Berg) Kiaersk.	Pitanga	pain, throat, flu, and kidneys	Infusion (I)	10	3	4	0.57
61.3. *Psidium guajava* L.	Goiabeira	diarrhea	Infusion (I)	19	1	1	0.17
61.4. *Psidium guineense* Sw.	Goiaba-áraça	pain, diarrhea, and hypertension	Infusion (I)	11	3	3	0.50
61.5. *Syzygium aromaticum* (L.) Merr. & L. M. Perry	Cravo-da-india	Throat and cough	Infusion (I)	5	1	2	0.23
61.6. *Syzygium jambolanum* (Lam.) DC.	Azeitona-preta	cholesterol	Decoction (I, E)	4	1	1	0.17
62. NYCTAGINACEAE							
62.1. *Boerhavia coccinea* L.	Amarra-pinto	bladder icterus, inflammation uterina, and kidneys	Infusion (I)	22	2	4	0.47
62.2. *Mirabilis jalapa* L.	Maravilha	heart, pain, and hypertension	Infusion (I)	8	2	3	0.40
63. OLACACEAE							
63.1. *Ximenia americana *L.	Limão-bravo	Trush and diuretic	Infusion (I)	4	2	2	0.33
64. OPILIACEAE							
64.1. *Agonandra brasiliensis* Miers ex Benth. & Hook f.	Pau-marfim	uterine inflammation	Decoction (I, E)	1	1	1	0.17
65. ORCHIDACEAE							
65.1. *Vanilla palmarum* (Salzm. ex Lindl.) Lindl.	Baunilha	hypertension	Infusion (I)	2	1	1	0.17
65.2. *Oncidium cebolleta *(Jacq.) Sw.	Orquidea	pain	Infusion (I)	2	1	1	0.17
66. OXALIDACEAE							
66.1. *Averrhoa carambola* L.	Carambola	hypertension	Infusion (I)	8	1	1	0.17
66.2. *Oxalis* aff. *hirsutissima* Mart. ex Zucc.	Azedinha	obesity	Infusion (I)	9	1	1	0.17
67. PAPAVERACEAE							
67.1. *Argemone mexicana* L.	Cardo-santo	hypertension	Infusion (I)	8	1	1	0.17
68. PASSIFLORACEAE							
68.1. *Passiflora alata* Curtis	Maracujá		Infusion (I)	9	1	1	0.17
68.2. *Passiflora cincinnata* Mast.	Maracujá-do-mato	soothing hypertension	Infusion (I)	5	2	2	0.33
69. PEDALIACEAE							
69.1* Sesamum indicum* L.	Gergelim	stomach, liver, gastritis, ulcer, and worms	Infusion and maceration (I)	12	2	5	0.53
70. PHYLLANTHACEAE							
70.1. *Phyllanthus niruri* L.	Quebra-pedra	kidneys	Infusion (I)	32	1	1	0.17
71. PHYTOLACCACEAE							
71.1. *Petiveria alliacea* L.	Guiné	rheumatism	Infusion (I, E)	4	1	1	0.17
72. PIPERACEAE							
72.1. *Piper callosum* Ruiz & Pav	Ventre-livre/elixir paregórico	kidneys	Infusion (I)	1	1	1	0.17
72.2. *Piper cuyabanum* C. DC.	Jaborandi	pain, stomach, and loss of hair	Infusion (I, E)	10	3	3	0.50
72.3. *Pothomorphe umbellata* (L.) Miq.	Pariparoba	blood cleanser, stomach, liver, and pneumonia	Infusion (I)	11	3	3	0.50
73. PLANTAGINACEAE							
73.1. *Plantago major* L.	Tanchagem	heart, pain, and laxative	Infusion (I)	16	3	3	0.50
74. POACEAE							
74.1. *Andropogon bicornis* L.	Capim-rabo-de-lobo	uterine inflammation	Infusion (I)	3	1	1	0.17
74.2. *Coix lacryma-jobi* L.	Lácrimas-de-nossa-senhora	kidneys	Infusion (I, E)	4	1	1	0.17
74.3. *Cymbopogon citratus* (DC.) Stapfc	Capim-cidreira	soothing blood cleanser, pain, stomach, expectorant, fever, flu, hypertension, muscular relaxative, kidneys, tachycardia, and cough	Infusion and juice (I)	49	5	12	1.30
74.4. *Cymbopogon nardus* (L.) Rendle.	Capim-citronela	flu, cough, and tuberculosis	Infusion (E)	11	2	2	0.33
*7*4.5. *Digitaria insularis* (L.) Mez ex Ekman	Capim-amargoso	wound healing, stomach, bone fractures, and rheumatism	Infusion (I)	14	3	4	0.57
*7*4.6. *Eleusine indica* (L.) Gaertn.	Capim-pé-de-galinha	Hypertension and swelling in pregnant woman	Infusion (I)	6	2	2	0.33
74.7. *Imperata brasiliensis* Trin.	Capim-sapé	diabetes, pain, hepatitis, kidneys, and vitiligo	Infusion (I)	12	5	5	0.83
74.8. *Melinis minutiflora* P. Beauv.	Capim-gordura	dengue, blood cleanser, stroke, flu, kidneys, sinusitis, cough, and tumors	Infusion (I)	31	7	8	1.23
74.9. *Oryza sativa* L.	Arroz	bladder	Infusion (I)	1	1	1	0.17
74.10. *Saccharum officinarum* L.	Cana-de-açúcar	kidneys, anemia, and hypertension	Infusion (I)	2	3	3	0.50
74.11. *Zea mays* L.	Milho	bladder kidneys	Infusion (I)	3	2	2	0.33
75. POLYGALACEAE							
75.1. *Polygala paniculata* L.	Bengué	rheumatism	Infusion (I)	6	1	1	0.17
76. POLYGONACEAE							
76.1. *Coccoloba cujabensis* Wedd.	Uveira	diuretic	Infusion (I)	1	1	1	0.17
76.2. *Polygonum* cf. *punctatum* Elliott	Erva-de-bicho	wound healing, dengue, stomach, fever, flu, and hemorrhoids	Infusion (I)	41	5	6	0.90
76.3. *Rheum palmatum* L.	Ruibarbo	blood cleanser, dysentery, pain, and snakebite	Infusion (I)	6	4	4	0.67
76.4. *Triplaris brasiliana* Cham.	Novatero	diabetes	Infusion (I)	1	1	1	0.17
77. POLYPODIACEAE							
77.1. *Phlebodium decumanum* (Willd.) J. Sm.	Rabo-de-macaco	diuretic, hepatitis, and kidneys	Infusion (I)	9	2	3	0.40
77.2. *Pteridium aquilinum* (L.) Kuhn	Samambaia	colic, blood cleanser, and rheumatism	Infusion (I)	8	3	3	0.50
77.3. *Pteridium* sp.	Samambaia-de-cipo	rheumatism	Infusion (I)	1	1	1	0.17
78. PONTEDERIACEAE							
*7*8.1. *Eichhornia azurea* (Sw.) Kunth	Aguapé	ulcer	Infusion (I)	3	1	1	0.17
79. PORTULACACEAE							
79.1. *Portulaca oleracea* L.	Onze-horas	hypertension	Infusion (I)	3	1	1	0.17
80. PROTEACEAE							
80.1. *Roupala montana* Aubl.	Carne-de-vaca	muscular relaxative	Infusion (I)	2	1	1	0.17
81. PUNICACEAE							
81.1. *Punica granatum* L.	Romã	colic, diarrhea, pain, throat, inflammation uterina, and kidneys	Infusion and maceration (I, E)	41	3	6	0.70
82. RHAMNACEAE							
82.1.* Rhamnidium elaeocarpum* Reissek	Cabriteiro	anemia, diarrhea, diuretic, pain, stomach, and worms	Infusion (I)	37	5	6	0.90
83. ROSACEAE							
83.1*. Rosa alba *L.	Rosa-branca	wound healing, pain, and uterine inflammation	Infusion and maceration (I, E)	6	3	3	0.50
83.2.* Rosa graciliflora* Rehder & E. H. Wilson	Rosa-amarela	pain	Infusion and maceration (I, E)	1	1	1	0.17
83.3. *Rubus brasiliensis *Mart.	Amoreira	cholesterol, hypertension, labyrinthitis, menopause, obesity, osteoporosis, and kidneys	Infusion and tintura (I)	38	6	7	1.07
84. RUBIACEAE							
84.1. *Chiococca alba *(L.) Hitchc.	Cainca	pain, flu, and rheumatism	Infusion (I)	8	3	3	0.50
84.2. *Cordiera edulis* (Rich.) Kuntze	Marmelada	worms	Maceration and syrup (I)	3	1	1	0.17
84.3. *Cordiera macrophylla* (K. Schum.) Kuntze	Marmelada-espinho	worms	Maceration and syrup (I)	1	1	1	0.17
84.4. *Cordiera sessilis* (Vell.) Kuntze	Marmelada-bola	Flu and worms	Maceration and syrup (I)	4	2	2	0.33
84.5. *Coutarea hexandra* (Jacq.) K. Schum.	Murtinha	diarrhea	Infusion (I)	1	1	1	0.17
84.6.* Genipa americana* L.	Jenipapo	appendicitis bronchitis diabetes and kidneys	Infusion and syrup (I)	8	4	4	0.67
84.7. *Guettarda viburnoides* Cham. & Schltdl.	Veludo-branco	blood cleanser and ulcer	Infusion (I)	5	2	2	0.33
84.8. *Palicourea coriacea* (Cham.) K. Schum.	Douradinha-do-campo	prostate cancer hearth, blood cleanser, diuretic, flu, hypertension, insomnia, relaxative muscular, and kidneys	Infusion (I)	62	7	9	1.30
84.9. *Palicourea rigida* Kunth	Doradão	Kidneys and cough	Infusion and decoction (I)	5	2	2	0.33
84.10. *Rudgea viburnoides* (Cham.) Benth.	Erva-molar	column, thooth, blood cleanser, dysentery, rheumatism, and kidneys	Infusion (I)	44	5	6	0.90
84.11. *Tocoyena formosa* (Cham. & Schltdl.) K. Schum.	Jenipapo-bravo	kidneys	Infusion (I)	1	1	1	0.17
84.12*. Uncaria tomentosa* (Willd. ex Roem. & Schult.) DC.	Unha-de-gato	intoxication, rheumatism, and kidneys	Infusion (I)	10	3	3	0.50
85. RUTACEAE							
85.1. *Citrus aurantiifolia* (Christm.) Swingle	Lima	soothing hearth, and hypertension	Infusion (I)	8	2	3	0.40
85.2. *Citrus limon* (L.) Osbeck	Limão	colic, diabetes, pain, liver, flu, hypertension, and cough	Infusion (I)	17	5	7	0.97
85.3. *Citrus sinensis* (L.) Osbeck	Laranja	soothing wound healing, fever, flu, pneumonia, and thyroid	Infusion (I)	30	4	6	0.80
85.4. *Ruta graveolens* L.	Arruda	colic, conjunctivitis, pain, stomach, fever, gastritis, nausea, and laxative muscular	Infusion (I)	57	4	8	0.93
85.5.* Spiranthera odoratissima* A.St.-Hil.	Manacá	rheumatism	Infusion (I)	6	1	1	0.17
85.6. *Zanthoxylum* cf. *rhoifolium* Lam.	Mamica-de-porca	diabetes, diarrhea, hemorrhoids, and muscular relaxative	Decoction (I, E)	12	4	4	0.67
86. SALICACEAE							
86.1. *Casearia silvestris *Sw.	Chá-de-frade	blood cleanser, pain, and fever	Infusion (I)	10	1	3	0.30
87. SAPINDACEAE							
87.1. *Dilodendron bipinnatum* Radlk.	Mulher-pobre	bone fractures	Infusion (I)	5	2	2	0.33
		uterine inflammation					
87.2. *Magonia pubescens* A. St.-Hil.	Timbó	wound healing, pain, and cough	Maceration (I, E)	7	2	3	0.40
87.3. *Serjania erecta* Radk.	Cinco-pontas	column, muscular relaxative, and kidneys	Infusion (I)	9	2	3	0.40
87.4. *Talisia esculenta* (A. St.-Hil.) Radlk.	Pitomba	column, pain, and rheumatism	Infusion (I)	6	2	3	0.40
88. SAPOTACEAE							
88.1. *Pouteria glomerata* (Miq.) Radlk.	Laranjinha-do-mato	fever	Infusion (I)	1	1	1	0.17
88.2. *Pouteria ramiflora* (Mart.) Radlk.	Fruta-de-viado	Ulcer and kidneys	Infusion (I)	1	2	2	0.33
89. SCROPHULARIACEAE							
89.1. *Bacopa* sp.	Vicki-de-batata	kidneys	Infusion (I)	2	1	1	0.17
89.2. *Scoparia dulcis* L.	Vassorinha	bladder wound healing, hearth, blood cleanser, diabetes, pain, bone fractures, swelling in pregnant woman, pneumonia, kidneys, syphilis, and cough	Infusion (I)	81	7	12	1.50
90. SIMAROUBACEAE							
90.1. *Simaba ferruginea* A. St.-Hil.	Calunga	anemia, wound healing, diabetes, digestive, pain, stomach, obesity, ulcer, and worms	Maceration (I)	31	7	9	1.30
90.2. *Simarouba versicolor* A. St.-Hil.	Pé-de-perdiz	wound healing and uterine inflammation	Decoction (I, E)	4	2	2	0.33
91. SIPARUNACEAE							
91.1. *Siparuna guianensis* Aubl.	Negramina	pain, fever, and flu	Infusion (I)	20	2	3	0.40
92. SMILACACEAE							
92.1*. Smilax* aff. *brasiliensis* Spreng.	Japecanga	Column and rheumatism	Infusion (I)	5	1	2	0.23
93. SOLANACEAE							
93.1*. Capsicum* sp.	Pimenta	Pain and hemorrhoids	Infusion (I, E)	14	2	2	0.33
93.2. *Nicotiana tabacum* L.	Fumo	thyroid	Infusion (I, E)	2	1	1	0.17
93.3. *Physalis* sp.	Tomate-de-capote	hepatitis	Infusion (I)	1	1	1	0.17
93.4.* Solanum americanum* Mill.	Maria-pretinha	worms	Infusion (I)	3	1	1	0.17
93.5. *Solanum lycocarpum* A. St.-Hil.	Fruta-de-lobo	Gastritis and ulcer	Infusion and maceration (I)	6	1	2	0.23
93.6.* Solanum* sp.	Jurubeba	column, stomach, and liver	Infusion (I)	8	2	3	0.40
93.7. *Solanum* sp.	Urtiga	boi	Infusion (I)	1	1	1	0.17
93.8. *Solanum melongena* L.	Berinjela	cholesterol	Infusion and maceration (I)	2	1	1	0.17
93.9. *Solanum tuberosum* L.	Batata-inglesa	Pain and gastritis	Infusion and maceration (I, E)	13	2	2	0.33
93.10. *Solanum viarum* Dunal.	Joá-manso	Hemorrhoids	Infusion (I)	7	1	1	0.17
94. TILIACEAE							
94.1. *Apeiba tibourbou *Aubl.	Jangadeira	liver	Decoction (I, E)	1	1	1	0.17
94.2. *Luehea divaricata* Mart.	Açoita-cavalo	uric acid, column, blood cleanser, throat, flu, hemorrhoids, intestine, pneumonia, muscular relaxative, kidneys, cough, and tumors	Decoction and syrup (I)	58	7	12	1.50
95. ULMACEAE							
95.1*. Trema micrantha* (L.) Blume	Piriquiteira	wound healing	Decoction (I, E)	1	1	1	0.17
96. VERBENACEAE							
96.1*. Casselia mansoi* Schau	Saúde-da-mulher	thooth, blood cleanser, uterine inflammation, and menstruation	Infusion (I)	9	3	4	0.57
96.2*. Duranta repens* L.	Pingo-de-ouro	diabetes	Infusion (I, E)	3	1	1	0.17
96.3. *Lantana camara *L.	Cambará	cold and cough	Decoction (I)	22	2	2	0.33
96.4. *Lippia alba* (Mill.) N. E. Br. ex Britton & P. Wilson	Erva-cidreira	soothing hearth, thooth, blood cleanser, pain, flu, hypertension, tachycardia, and cough	Infusion (I)	75	5	9	1.10
96.5. *Phyla* sp.	Chá-mineiro	conjunctivitis, blood cleanser, pain, fever, muscular relaxative, rheumatism, and kidneys	Infusion (I)	19	4	7	0.87
96.6. *Priva lappulacea* (L.) Pers.	Pega-pega	Stomach and sinusitis	Infusion (I)	2	2	2	0.33
96.7.* Stachytarpheta* aff. *cayennensis* (Rich.) Vahl	Gervão	bronchitis blood cleanser, stomach, liver, bone fractures, gastritis, flu, constipation, relaxative muscular, cough, and worms	Infusion (I)	80	6	11	1.33
96.8. *Stachytarpheta* sp.	Rabo-de-pavão	relaxative muscular	Infusion (I)	3	1	1	0.17
96.9. *Vitex cymosa* Bert.ex Spregn.	Tarumeiro	blood cleanser, diarrhea, pain, and stomach	Infusion (I)	8	3	4	0.57
97. VIOLACEAE							
97.1. *Anchietea salutaris* A. St.-Hil.	Cipó-suma	column, blood cleanser, fever, intoxication, and vitiligo	Infusion (I)	18	4	5	0.73
97.2. *Hybanthus calceolaria* (L.) Schulze-Menz.	Poaia-branca	cough	Infusion (I)	1	1	1	0.17
98. VITACEAE							
98.1. *Cissus cissyoides* L.	Insulina-de-ramo	diabetes	Infusion (I)	10	1	1	0.17
98.2. *Cissus gongylodes* Burch. ex Baker	Cipó-de-arráia	relaxative muscular	Infusion (I)	1	1	1	0.17
98.3. *Cissus* sp.	Rabo-de-arráia	hypertension	Infusion (I)	3	2	2	0.33
98.4.* Cissus* sp.	Sofre-do-rim-quem-quer	inflammation uterina, relaxative muscular, and kidneys	Infusion (I)	5	3	3	0.50
99. VOCHYSIACEAE							
99.1.* Callisthene fasciculata* Mart.	Carvão-branco	Hepatitis and icterus	Decoction (I, E)	10	2	2	0.33
99.2.* Qualea grandiflora* Mart.	Pau-terra	Diarrhea and pain	Decoction (I, E)	5	2	2	0.33
99.3.* Qualea parviflora* Mart.	Pau-terrinha	diarrhea		1	1	1	0.17
99.4.* Salvertia convallariodora* A. St.-Hil.	Capotão	diarrhea, diuretic, hemorrhoids, and relaxative muscular	Decoction (I, E)	4	4	4	0.67
99.5.* Vochysia cinnamomea *Pohl	Quina-doce	flu		3	1	1	0.17
99.6.* Vochysia rufa* Mart.	Pau-doce	blood cleanser, diabetes, diarrhea, laxative, obesity, kidneys, cough, and worms	Decoction, Infusion (I, E)	25	6	8	1.13
100. LILIACEAE							
100.1.* Aloe barbadensis* Mill.	Babosa	Cancer, prostate cancer, wound healing, diabetes, stomach, bone fractures, gastritis, hepatitis, laxative, and rheumatism	Syrup and maceration (I, E)	87	5	9	1.10
101. ZAMIACEAE							
101.1. *Zamia boliviana* (Brongn.) A. DC.	Maquiné	stomach	Infusion (I)	2	1	1	0.17
102. ZINGIBERACEAE							
102.1.* Alpinia speciosa* (J. C. Wendl.) K. Schum.	Colônia	soothing hearth, fever, flu, and hypertension	Infusion (I)	36	4	5	0.73
102.2. *Curcuma longa* L.	Açafrão	column, diuretic, pain, stomach, and hepatitis	Infusion and maceration (I)	18	4	5	0.73
102.3. *Zingiber officinale* Roscoe	Gengibre	pain, flu, sinusitis, and cough	Infusion and maceration (I)	26	2	4	0.47

I: Internal, E: External; NSC: Number of body systems treated by species; NCS: number of body systems. NP: Number of properties of the species; RI: Relative importance of the species.

**Table 4:** Species with the highest values of relative importance.

Family	Species	Application/citation	RF	RI
Apocynaceae	* Himatanthus obovatus* (Müll. Arg.) Woodson	anemia (1), wound healing (7), cholesterol (3), blood cleanser (9), pain (4), nose bleeding (1), hypertension (4), uterine inflammation (5), labyrinthitis (6), muscle relaxant (2), worms (1), vitiligo (1), and pneumonia (1)	45	1.87
Malvaceae	* Hibiscus sabdariffa* L	anxiety/heart (1), flu (1), tachycardia (1), kidneys (1), cramps (3), discharge (1), diarrhea (1), pain (1), inflammation uterine (2), labyrinthitis (3), snakebite (1), and pneumonia (2)	18	1.87
Asteraceae	* Solidago microglossa *DC.	wound healing (53), blood cleanser (11), pain (2), bone fractures (1), hypertension (1), uterine inflammation (3), muscle relaxant (6), kidneys (3), and worms (2)	82	1.8
Loganiaceae	* Strychnos pseudoquina *A. St.-Hil.	anemia (46), wound healing (3), cholesterol (1), blood cleanser (16), pain (13), stomach (3), bone fractures (1), flu (2), uterine inflammation (1), pneumonia (1), muscle relaxant (1), cough (10), ulcer (1), and worms (8)	107	1.73
Moraceae	* Dorstenia brasiliensis* Lam.	wound healing (1), colic (1), tooth ache (1), blood cleanser (4), dysentery (1), pain (7), flu (2), laxative (3), menstruation (1), pneumonia (6), relapse delivery (13), and kidneys (1)	41	1.5
Plantaginaceae	* Scoparia dulcis* L.	heart (6), blood cleanser (1), diabetes (1), pain (16), bone fractures (47), swelling in pregnant woman (4), pneumonia (1), kidneys, ( 1) syphilis (3), and cough (1)	55	1.5
Malvaceae	* Luehea divaricata *Mart.	uric acid (18), vertebral column (2), blood cleanser (1), throat (1), flu (1), hemorrhoids (7), intestine (1), pneumonia (8), muscle relaxant (2), kidneys (3), cough (10), and tumors (4)	58	1.5

RF: Relative frequency; RI: Relative importance of the species.

**Table 5:** Categories of diseases, indications, form of use, preparation and the informant consensus factor of the main medicinal plants from Nossa Senhora Aparecida do Chumbo District, Poconé, Mato Grosso, Brazil.

Disease category/CID, 10th ed.	Medicinal plants	Main indications	Main forms of use	Part utilized/ State of the plant	Species/citations	ICF
Injuries, poisoning, and certain other consequences of external causes— XIX	*Scoparia dulcis* L.* Solidago microglossa *D. C* Lafoensia pacari* A. St.-Hil.	inflammation and pain	Inf, Dec, Mac, and Tin	L, Wp, Rt (Fr, Dr)	65/286	0.78
Mental and behavioural disorders —V	*Chamomilla recutita* (L.) Rauschert.	soothing	Dec and Inf	L (In, Sc)	20/85	0.77
Symptoms, signs, and abnormal clinical and laboratory findings not elsewhere classified—XVIII	*Macrosiphonia longiflora* (Desf.) Müll. Arg.	blood depurative	Inf, Dec, and Mac	Rz (Fr, Dr)	176/713	0.75
Diseases of the genitourinary system —XIV	*Palicourea coriacea* (Cham.) K. Schum.	Kidneys and diuretic	Inf, Dec, and Syr	L (Fr, Dr)	132/533	0.75
Diseases of the digestive system—XI	*Plectranthus barbatus* Andrews	stomach, pain, liver, and malaise	Dec, Inf, Mac, and Juc	L (Fr, Dr)	113/428	0.74
Other infectious and parasitic diseases—I	*Chenopodium ambrosioides* L.	verminose	Inf, Mac, and Juc	L (Fr,Dr)	82/300	0.73
Diseases of the respiratory system—X	*Mentha pulegium* L.	flu, bronchitis, colds, and cough	Dec, Inf, Mac, and Syr	L (Fr, Dr)	88/303	0.71
Pregnancy, childbirth, and the puerperium—XV	*Dorstenia brasiliensis* Lam.	childbirth	Dec, Inf, and Syr	Rz (Fr, Dr)	9/28	0.70
Diseases of the circulatory system—IX	*Alpinia speciosa* (J. C. Wendl.) K. Schum.	Hypertension and heart	Inf and Mac	L (Fr, Dr)	56/180	0.69
Some disorders originating in the perinatal period—XVI	*Bidens pilosa* L.	Hepatitis and enteric	Dec and Inf	L (In, Sc)	3/7	0.67
Diseases of blood and blood forming organs and certain disorders involving the immune system—III	*Strychnos pseudoquina* A. St.-Hil.	anemia	Inf, Mac, and Syr	B (Fr, Dr)	15/38	0.62
Diseases of the eye and the surrounding structures—VII	*Malva sylvestris* L.	Discharge and conjuctivitis	Inf and Tin	L (Fr, Dr)	6/14	0.61
Diseases of endocrine of nutritional and metabolic origins—IV	*Cissus cissyoides* L.	diabetes	Inf	L (Fr, Dr)	47/109	0.57
Diseases of the ear and mastoid process—VIII	*Himatanthus obovatus *(Müll. Arg.) Woodson	labyrinthitis	Inf	L (Fr, Dr)	7/15	0.57
Diseases of musculoskeletal and connective tissue—XIII	*Solidago microglossa* DC.	bone fractures	Dec, Inf, Mac, and Tin	L (Fr, Dr)	70/146	0.52
Diseases of the skin and subcutaneous tissue—XII	*Dioscorea brasiliensis* Willd.	furuncules	Dec, Inf, Mac, Tin, and Out	Rz (Fr, Dr)	29/51	0.44
Neoplasia (tumors)—II	*Aloe barbadensis* Mill.	wound healing	Dec, Inf, Mac, Tin, and Out	L (Fr, Dr)	22/38	0.43
Diseases of the nervous system—VI	*Macrosiphonia longiflora* (Desf.) Müll. Arg.	leakage	Inf		14/16	0.13

CID, 10th ed. categories of diseases in chapters according to International Classification of Diseases and Related Health Problems, 10th. edition [25]; ICF: informant consesus factor; Inf: infusion, Dec: decoction, Syr: syrup, Mac: maceration, Sal: salad, Tin: tinture, Juc: juice, Out: others (compression and bath). L: leave; Wp: whole plant; Rt: root; Rz: rhizome; B: bark. State of the plant: Fr: fresh; Dr: dried.
